# Human iPSC-derived alveolar macrophages reveal macrophage subtype functions of itaconate in *M*. *tuberculosis* defense

**DOI:** 10.1172/jci.insight.198342

**Published:** 2026-03-09

**Authors:** Adam S. Krebs, Tomi Lazarov, Anthony T. Reynolds, Kimberly A. Dill-McFarland, Abigail Xie, James M. Bean, Muxue Du, Olivier Levy, John A. Buglino, Aaron Zhong, Anna-Lena Neehus, Stéphanie Boisson-Dupuis, Jean-Laurent Casanova, Elouise E. Kroon, Marlo Möller, Thomas R. Hawn, Ting Zhou, Lydia W.S. Finley, Marc Antoine Jean Juste, Dan W. Fitzgerald, Frederic Geissmann, Michael S. Glickman

**Affiliations:** 1 Immunology Program, Sloan Kettering Institute, Memorial Sloan Kettering Cancer Center, New York, New York, USA.; 2 Immunology and Microbial Pathogenesis Program, Weill Cornell Medicine, New York, New York, USA.; 3 Department of Medicine, University of Washington, Seattle, Washington, USA.; 4 Cell Biology Program, Sloan Kettering Institute, New York, New York, USA.; 5 Weill Cornell Graduate School of Medical Sciences, Cornell University, New York, New York, USA.; 6 The SKI Stem Cell Research Facility, The Center for Stem Cell Biology and Developmental Biology Program, Sloan-Kettering Institute, New York, New York, USA.; 7 Rockefeller University, New York, New York, USA.; 8 South African Medical Research Council Centre for Tuberculosis Research, Division of Molecular Biology and Human Genetics, Faculty of Medicine and Health Sciences, Stellenbosch University, Cape Town, South Africa.; 9 Centre for Bioinformatics and Computational Biology, Stellenbosch University, Cape Town, South Africa.; 10 National Institute for Theoretical and Computational Sciences (NITheCS) South Africa.; 11 Genomics for Health in Africa (GHA), Africa-Europe Cluster of Research Excellence (CoRE), Ghana.; 12 GHESKIO Center, Port Au Prince, Haiti.; 13 Center for Global Health, Weill Cornell Medicine, New York, New York, USA.

**Keywords:** Immunology, Infectious disease, Pulmonology, Cytokines, Macrophages, Tuberculosis

## Abstract

*Mycobacterium tuberculosis* (Mtb) survives within multiple macrophage populations during infection, including alveolar macrophages (AMs) and recruited inflammatory macrophages. In mice, itaconate, produced in macrophages by ACOD1-mediated decarboxylation of aconitate, has direct antimicrobial activity, modulates inflammatory cytokines, and is required for resistance to Mtb infection. The role of itaconate in human macrophages is less clear, and it is unknown whether itaconate mediates distinct effects in macrophage subtypes. Here, we investigated the role of itaconate in macrophages derived from human induced pluripotent stem cells (iPSCs), induced by either GM-CSF to resemble AMs (AM-like cells, hereafter ipAM-Ls) or M-CSF to resemble monocyte-derived macrophages (MDM-like cells, hereafter ipMDM-Ls). Both human macrophage types produced substantially less itaconate than mouse macrophages, and ipAM-Ls produced 4-fold less itaconate than ipMDM-Ls. Surprisingly, *ACOD1*-deficient ipAM-Ls, but not ipMDM-Ls, were permissive for Mtb growth. Moreover, itaconate functioned to dampen the Mtb-induced inflammatory response in ipMDM-Ls, but not ipAM-Ls, affecting both the type I IFN and TNF pathways. These results indicate that itaconate is involved in human macrophage responses to tuberculosis, with distinct roles in different macrophage subsets. These results also show that genetically tractable iPSC-derived macrophages are a useful model to dissect cellular host-pathogen interactions in human macrophages.

## Abstract 2

In this study the authors develop a stem cell derived lung alveolar macrophage model and use these cells to dissect the contribution of the inflammation induced metabolite itaconate in defense against *M. tuberculosis* infection. The findings show that the function of itaconate in host defense differs between human macrophage types.

## Introduction

*Mycobacterium tuberculosis* (Mtb), the causative agent of tuberculosis, is a human-specific pathogen that has spent millennia adapting to a cascade of immunological events to ultimately evade host control and facilitate bacterial dissemination and survival (1, 2). Mtb infection is initiated when droplets from the lung of an infected individual are inhaled and deposited in the terminal alveolus of the lung. Tissue-resident alveolar macrophages (AMs) are generally understood to be the initial host cell for Mtb, a model supported by mouse infection research showing AMs to be the predominant infected cell at early time points after infection (3). With progressive infection, bacteria infect additional leukocyte populations recruited from the blood, including neutrophils, monocyte-derived macrophages (MDMs), and interstitial macrophages (3–5). These sequential cellular hosts apply distinct pressure to Mtb*,* suggesting that the pathogen must rapidly adapt to distinct effector mechanisms in macrophage subtypes (5, 6).

AMs are a distinct macrophage population that reside in lung alveoli. AMs are self-renewing tissue macrophages, largely independent from circulating monocytes (7, 8), that develop during embryonic development (9–15). A hallmark feature of mouse and human AMs is the dependence on GM-CSF for the induction of the transcription factor PPARγ (PPARG), which supports their development, maintenance, and function within the pulmonary environment (16–19). Both PPARG- and GM-CSF–deficient mice, as well as humans with genetic GM-CSF receptor deficiency or neutralizing autoantibodies against GM-CSF, experience alveolar proteinosis due to absent/defective AMs (16, 20–24). AMs have an antiinflammatory function in pulmonary immunity via the rapid clearance of cellular debris and pathogens (15, 25, 26). Through the study of macrophage dynamics in Mtb-infected murine lungs and AMs recovered from healthy human lungs infected ex vivo with pulmonary pathogens like Mtb and influenza A virus, AMs and MDMs have been found to have distinct transcriptional responses to pathogens (27–30). Studies of Mtb infection of AMs obtained by bronchoalveolar lavage suggest that there is a large interindividual variability (31), but AMs are generally understood to be hypoinflammatory in response to pathogen infection (32). Our incomplete knowledge of Mtb-AM infection dynamics is in part due to the challenge of studying AMs from humans, including the near impossibility of obtaining these lung-resident cells during the clinically silent period after initial Mtb infection and the genetic intractability of primary human AMs.

Itaconic acid is a macrophage product implicated in macrophage-Mtb interactions. This metabolite is produced by inflammatory induction of the enzyme aconitate decarboxylase 1 (ACOD1, encoded by *ACOD1*, previously named *IRG1* in the literature), which decarboxylates cis-aconitate to produce itaconate (33). Itaconate has complex effects on inflammation and bacterial control, including immunomodulatory effects through its inhibition of succinate dehydrogenase (SDH), prevention of IL-1β maturation, and modification of IκBζ-mediated inflammatory transcription (34–37). Itaconate can also directly inhibit bacterial growth through inhibition of bacterial isocitrate lyase and methyl-malonyl-CoA mutase (MCM), and may thereby be a direct antibacterial effector of macrophages (38). *ACOD1*^–/–^ mice, which cannot produce itaconate, are highly susceptible to Mtb aerosol infection due to severe pulmonary inflammation (39). Murine *ACOD1*^–/–^BMDMs overexpress genes in the TNF pathway with tuberculosis infection, but do not have a cell-intrinsic defect in controlling bacterial replication, suggesting that the predominant role of itaconate in Mtb infection is the regulation of myeloid inflammatory responses rather than direct bacterial killing (39).

Murine macrophages produce very high levels of itaconate, but the role of itaconate in human macrophages is less well understood. In vitro and transfection experiments show that human ACOD1 is catalytically less active than murine ACOD1, suggesting that human macrophages may produce less itaconate (40). Although RAW264.7 cells, a commonly used murine macrophage cell line, produce approximately 8 mM itaconate upon stimulation with LPS, human PBMC-derived macrophages produce approximately 60 μM under similar conditions (41). Beyond this difference, it is unknown whether macrophages of distinct ontogeny (i.e., tissue resident vs. blood derived) differ in production of itaconate and its role in immunomodulation and antibacterial activities.

To address these questions, we developed a genetically tractable human induced pluripotent stem cell (iPSC) model of human macrophage differentiation (42–45) that can be used to produce alveolar-like macrophages (ipAM-Ls) that resemble their bona fide human AM counterparts, and syngeneic M-CSF–derived macrophages (ipMDM-Ls) as controls. We next used this model to measure and compare itaconate production in these cells, their inflammatory response and permissiveness to Mtb, and the role of itaconate by generating ACOD1-deficient and isogenic control human iPSC lines. Our results showed that itaconate is involved in the response of human macrophages to Mtb, despite a lower level of production in humans in comparison to mice; revealed that itaconate has different functions in ipAM-L versus ipMDM-L cells; and established human iPSC-derived macrophage subtypes as a genetically tractable host-cell model system to study the pathogenesis of Mtb.

## Results

### Generation of human ipAM-Ls through hematopoietic differentiation of peripheral blood–derived iPSCs.

To develop a system for generating human alveolar-like and control macrophages, we utilized GM-CSF or M-CSF, respectively, for terminal differentiation of myeloid progenitor cells produced through hematopoietic differentiation of iPSCs (46). Embryoid bodies generated from human iPSC clusters were matured for 14 days in media containing human IL-3 and M-CSF. Mature embryoid bodies produced nonadherent myeloid progenitor cells that were terminally differentiated into distinct macrophage subtypes with GM-CSF or M-CSF, which we label as ipAM-L (alveolar-like) or ipMDM-L (M-CSF–derived macrophages), respectively (Supplemental Figure 1A; supplemental material available online with this article; https://doi.org/10.1172/jci.insight.198342DS1). ipAM-L cells survived in the absence of M-CSF (data not shown), which is consistent with the in vivo observation that AMs are found in M-CSF–deficient mice (47–49).

**Figure 1:**
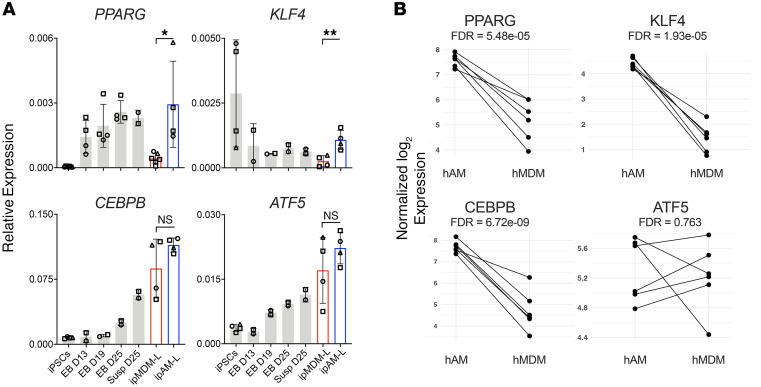
AM-defining transcription factors are expressed in ipAM-L cells. (**A**) RT-qPCR assessment of mRNAs encoding transcription factors that define tissue-resident alveolar macrophages (PPARγ, KLF4, CEBPB, ATF5) at each stage of the iPSC differentiation process (see Supplemental Figure 1A), normalized to GAPDH. Data are derived from 3 independent iPSC donors, each signified as a different symbol. Unpaired, 2-tailed *t* test used to assess for significant differences between ipMDM-L and ipAM-L populations; ns, not significant; *P < 0.05, **P < 0.01. (**B**) RNA levels encoding the transcription factors PPARγ, KLF4, CEBPB, and ATF5 in primary AMs isolated by bronchoalveolar lavage (hAM) and monocyte-derived macrophages (hMDMs) derived from reanalysis of RNA-Seq data published in Campo et al. (27).

Brightfield imaging and May-Grunwald Giemsa staining confirmed that both the ipMDM-L and ipAM-L cells resembled macrophages based on the large, irregular shape, well-defined dark nucleus, and pale pink to purple cytosol with prominent vacuoles (Supplemental Figure 1B). To determine whether ipAM-L and ipMDM-L cells differ in the expression of transcription factors characteristic of AMs in the human lung (Supplemental Figure 2A), we measured the mRNAs encoding PPARG, KLF4, CEBPB, and ATF5 (18). ipAM-L cells had significantly higher levels of PPARG and KLF4 compared with ipMDM-L cells (Figure 1A). ipAM-L cells also had a trend toward higher relative expression of CEBPB and ATF5 compared with ipMDM-L cells (Figure 1A). To assess the homogeneity of these iPSC-derived macrophage populations and to compare their surface marker expression with that of AMs in the human lung (Supplemental Figure 2A), we stained ipAM-L and ipMDM-L cells for MRC1, Dectin 1, HLA-DR, CD14, and CD11c. We observed homogenous populations of ipAM-L cells expressing higher levels of MRC1, Dectin 1, HLA-DR, and CD14, consistent with the surface phenotype of AMs (Supplemental Figure 2, B and C).

**Figure 2:**
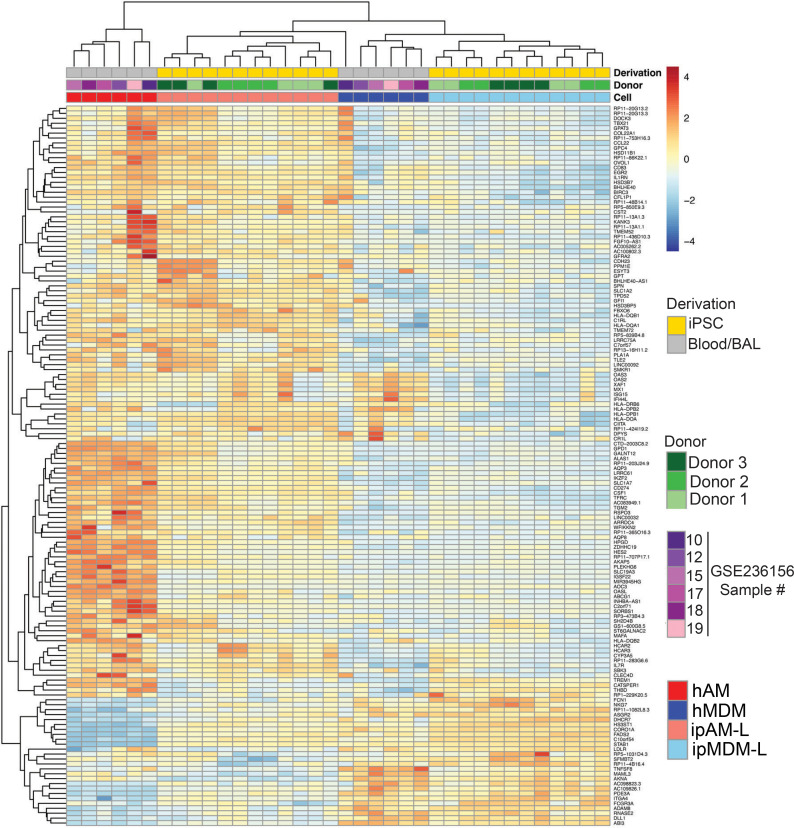
ipAM-Ls and ipMDM-Ls resemble human alveolar and monocyte-derived macrophages in the basal transcriptional state. The heatmap clusters the 146 most significant differentially expressed genes between iPSC-derived GM-CSF (ipAM-L) and M-CSF (ipMDM-L) cells from 3 donors (green shades) and in 6 donors (purple shades) profiled in Campo et al. (27). Also see Supplemental Figure 3 for an expanded 736 gene set.

To further interrogate the similarity of these iPSC-derived macrophage types to human AMs and MDMs, we compared transcriptional profiles of iPSC macrophages to a published dataset of AMs collected by bronchoalveolar lavage from 6 healthy donors with paired MDMs from the same donors (27). Analysis of these AM-MDM pairs confirmed upregulation PPARG, KLF4, and CEBPB in AMs (Figure 1B). To more thoroughly compare iPSC-derived ipAM-Ls and ipMDM-Ls to these primary human macrophages, we derived iPSCs from 3 donors and subsequently produced macrophages to perform RNA-Seq and compared these data to the ex vivo AMs versus MDMs. Using the 146 (Figure 2) or 736 (Supplemental Figure 3) most differentially expressed genes (DEGs) between iPSC-derived ipMDM-L and ipAM-L cells, we clustered gene expression and observed clear clustering of primary bronchoalveolar lavage–isolated AMs with ipAM-Ls and of MDMs with ipMDM-Ls (Figure 2 and Supplemental Figure 3). Taken together, these data indicate that GM-CSF–dependent differentiation of embryoid body–derived human macrophages results in a gene expression profile that resembles tissue-resident AMs, and that M-CSF–dependent differentiation of embryoid body–derived human macrophages resembles MDMs.

### ipAM-L cells transcriptionally resemble bona fide AMs with Mtb infection.

To assess whether human iPSC-derived macrophages resemble their ex vivo AM and MDM counterparts during Mtb infection, we performed RNA-Seq of ipAM-L and ipMDM-L cells infected with Mtb. These transcriptomic profiles were compared against 2 transcriptomic datasets of Mtb-infected macrophages (27, 42). The dataset in Campo et al. was used as a reference since it evaluates the response of primary human AMs isolated by bronchioalveolar lavage to Mtb (27). The Arias et al. dataset was generated from Mtb-infected alveolar-like macrophages differentiated from PBMCs by GM-CSF, TGF-β, and synthetic surfactant (termed here blAM-L) (50) (see Table 1 for macrophage nomenclature for all populations).

With Mtb infection, primary human AMs and blAM-L macrophages had a higher number of DEGs than iPSC-derived macrophages (Supplemental Figure 4). AMs and blAM-L macrophages had 400 DEGs that changed in the same direction in response to Mtb, whereas 154 DEGs underwent transcriptional changes in the opposite direction (Supplemental Figure 4). In contrast, all 69 DEGs shared between the AMs and iPSC-derived AM-L macrophages changed in the same direction (Supplemental Figure 4). blAM-L cells were the most transcriptionally reactive to Mtb infection and, based on the number of overlapping DEGs, more closely resembled ipMDM-L cells rather than ipAM-L cells.

**Figure 3:**
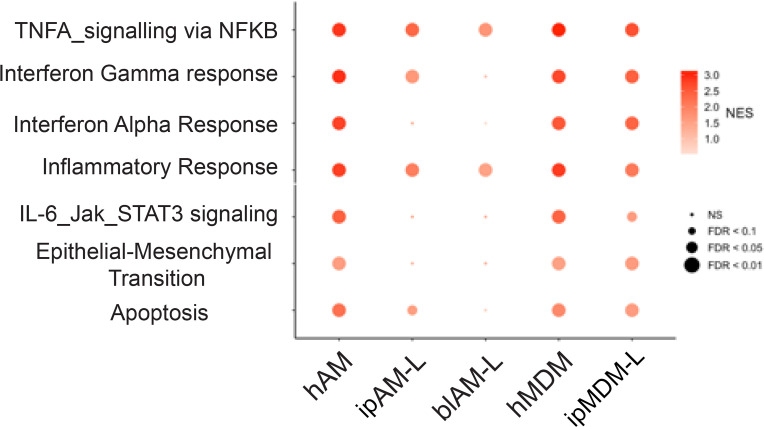
Conserved response of macrophage types to Mtb infection. Normalized enrichment score (NES) plotted for each hallmark pathway induced by Mtb infection for each macrophage type listed in Table 1.

To better characterize the transcriptional responses of these macrophage populations to Mtb infection, pathway enrichment analysis was conducted. Mtb infection induced significant enrichment of genes in the TNF-α signaling pathway by NF-κB and inflammatory response pathways in all macrophage populations assessed (Figure 3). Genes in the IFN-γ response and apoptosis pathways were also upregulated in all macrophage types except for blAM-L macrophages (Figure 3). Comparison of the transcriptional response of Mtb-infected MDMs and ipMDM-Ls revealed highly similar pathway enrichment (Figure 3). Taken together, these data indicate that the transcriptional responses of ipAM-L, ipMDM-L, and blAM-L cells to Mtb resemble those of primary human macrophage counterparts.

### Conserved response of iPSC-derived macrophages to Mtb infection across donors.

**Figure 4:**
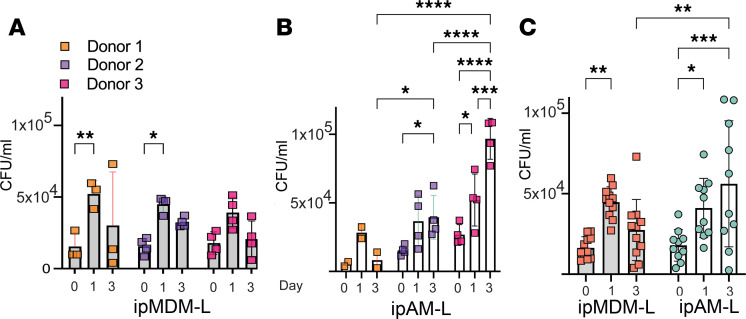
ipAM-Ls are permissive for tuberculosis growth. ipMDM-Ls (**A**) and ipAM-Ls (**B**) from 3 iPSC donors were infected with Mtb at a target MOI of 3. CFUs were quantified immediately after infection and 1 and 3 days after infection. Each donor is depicted with a different color. (**C**) Combined data from all donors for ipMDM-Ls (shaded bar) and ipAM-Ls (clear bar). Statistical significance in all panels is by 2-way ANOVA with correction for multiple comparisons. *P < 0.05, **P < 0.01, ***P <.0001, ****P < 0.00001.

Although iPSCs and the resulting macrophages derived from different donors will clearly differ due to genetic heterogeneity, to serve as a model of Mtb infection, these cells should share a core response to Mtb infection independent of donor source and differentiation instance. To assess the inter-donor and differentiation variability of the iPSC macrophage model, we differentiated ipAM-Ls and ipMDM-Ls from 3 donors. Macrophages from all donors had comparable uptake of Mtb, and ipMDM-L cells from all donors were generally restrictive of Mtb growth such that bacterial titers did not rise substantially over the input (Figure 4A). In contrast, ipAM-L cells were more permissive for Mtb growth, with bacterial titers rising on day 1 in all donors and further by day 3 in donors 2 and 3, with donor 3 being the most permissive (Figure 4B). Aggregate analysis across donors confirmed that ipAM-L cells are more permissive for tuberculosis growth than ipMDM-L cells (Figure 4C).

**Figure 5:**
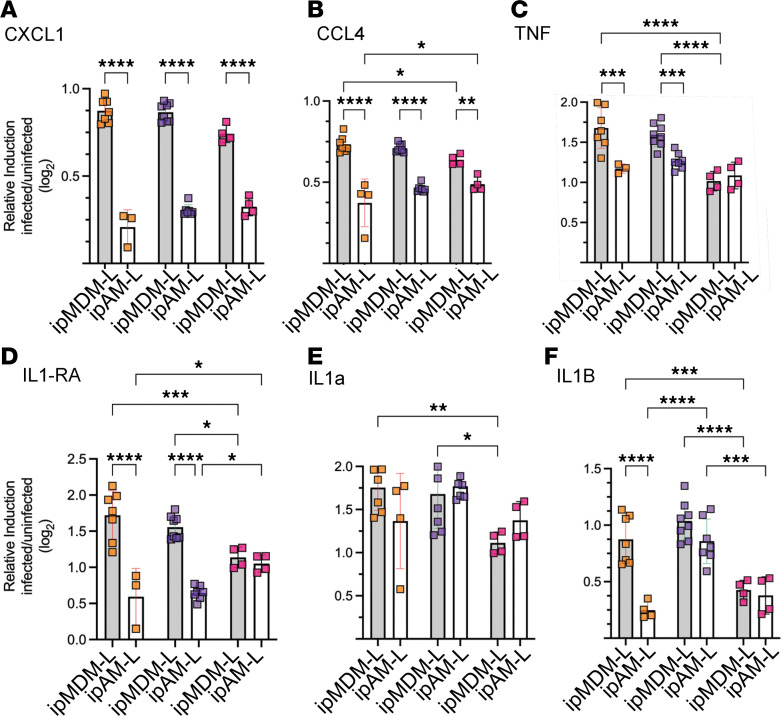
ipAM-L cells are hypoinflammatory with Mtb infection. Cytokine and chemokine responses of ipMDM-L or ipAM-L cells assayed on day 3 of Mtb infection. In each graph, ipMDM-L cells are the shaded bars and ipAM-L cells are clear bars, with donor identity as depicted in Figure 4A. Each *y* axis is the log_2_ fold-change of the indicated cytokine of infected/uninfected determined by Luminex (see Methods) for CXCL-1 (**A**), CCL4 (**B**), TNF (**C**), IL-1RA (**D**) IL-1α (**E**), and IL-1β (**F**). For all panels, 2-way ANOVA was used to assess variance of the samples based on infection status and within a macrophage population. *P < 0.05, **P < 0.01, ***P < 0.0001, ****P < 0.00001.

To assess the conservation of the inflammatory response of ipAM-L and ipMDM-L cells to Mtb infection, we measured cytokine and chemokine levels in the supernatants of infected macrophages. Mtb infection induced all measured chemokines and cytokines, regardless of macrophage subtype or donor (Figures 5, A–F), with ipAM-L cells hypoinflammatory for most cytokines, including CXCL1 (Figure 5A), CCL4 (Figure 5B), and TNF (Figure 5C). We observed some inter-donor heterogeneity, including donor-specific differences in IL-1RA (Figure 5D), IL-1α (Figure 5E), and IL-1β (Figure 5F). Overall, these data indicate that ipAM-L and ipMDM-L cells consistently respond to Mtb infection largely independent of iPSC source and that independent derivations from distinct iPSC sources produce macrophages with conserved responses to Mtb infection.

### ipAM-L and ipMDM-L cells differ in itaconate production.

**Figure 6:**
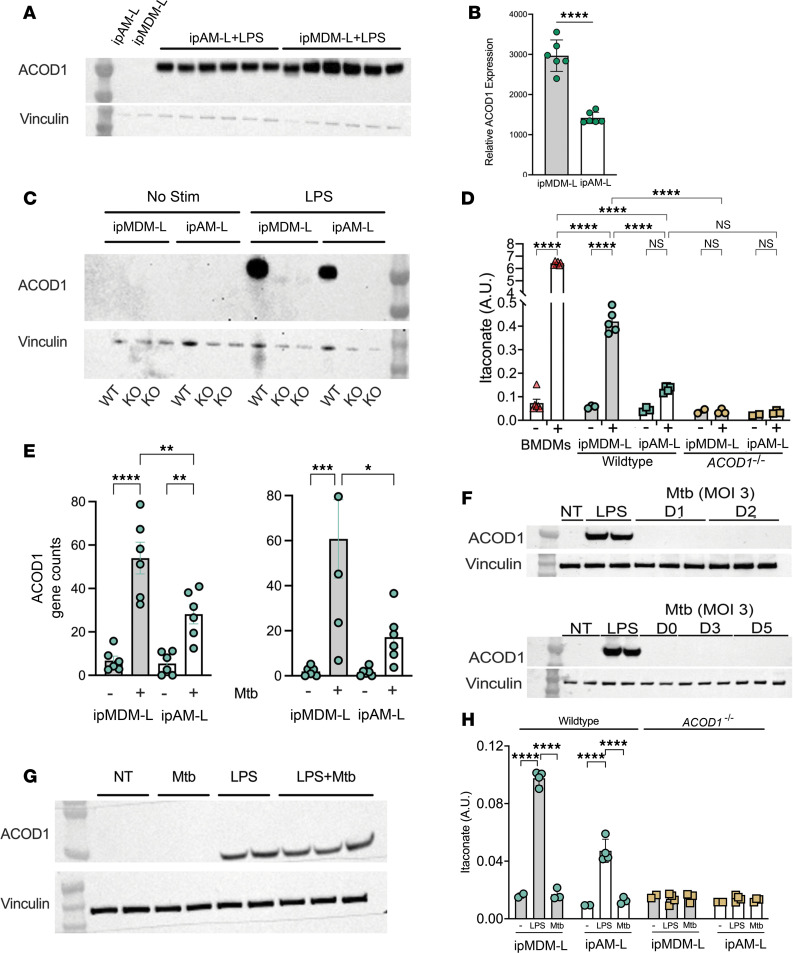
Human macrophages are feeble producers of itaconate with Mtb infection. (**A** and **B**) Expression of ACOD1 protein in iPSC-derived macrophage subtypes. Anti-ACOD1/Vinculin immunoblot of ipMDM-L or ipAM-L cells after 6 hours of stimulation with LPS (100 ng/mL) (**A**) and normalized quantitation of ACOD1 protein levels (**B**). ****P < 0.0001 by unpaired, 2-tailed *t* test.(**C**) Production of *ACOD1^–/–^* macrophages. ipMDM-Ls and ipAM-Ls were differentiated from CRISPR-edited iPSCs (see Supplemental Figure 5) or isogenic control cells. Anti-ACOD1 immunoblot of ipMDM-L and ipAM-L cells derived from *ACOD1-*deficient iPSCs or isogenic controls stimulated with LPS as in **A** and **B**. (**D**) Human macrophage production of itaconate. GC-MS quantification of itaconate produced by mouse BMDMs, ipMDM-Ls, ipAM-Ls, or *ACOD1^–/–^* ipMDM-Ls and ipAM-Ls without (–) or with (+) 6 hours of LPS stimulation. Itaconate levels are graphed AU by normalization to total protein levels; 2-way ANOVA used to assess variance of the samples within the same treatment group and based on stimulation status. ****P < 0.0001. (**E**) Gene counts of *ACOD1* mRNA after 4 hours or 24 hours without (–) or with (+) Mtb infection of ipMDM-Ls or ipAM-Ls. Data derived from 3 donors in biological duplicates. ***P < 0.005 ****P < 0.0001 by 2-way ANOVA. (**F**) Anti-ACOD1/Vinculin immunoblot of WT ipMDM-L cells after no treatment (NT), LPS stimulation (100 ng/mL), or Mtb infection at MOI of 3 and assayed at days 1 and 2 (top), or days 0, 3, and 5 (bottom). (**G**) Anti-ACOD1/Vinculin immunoblot of WT ipMDM-L cells after no treatment (NT), 4 hours of LPS stimulation (100 ng/mL), or infection with Mtb at MOI of 10 for 4 hours, or both. (**H**) GC-MS quantification of itaconate produced by ipMDM-Ls and ipAM-Ls after 4 hours of infection with Mtb (MOI 10) or LPS (100 ng/mL). Itaconate levels were normalized to total protein levels.

Having established a human iPSC-derived macrophage system to study Mtb-macrophage interactions in distinct macrophage types, we sought to use this system to understand the role of itaconate in human macrophages generally and AMs specifically. We first evaluated the expression of ACOD1, encoded by *ACOD1*, in ipMDM-L and ipAM-L cells by stimulation with 100 ng/mL of LPS and immunoblotting using an anti-ACOD1 antibody. ACOD1 was not detected in unstimulated macrophages of either type (Figure 6A), but LPS stimulation strongly induced ACOD1 protein expression. ipMDM-Ls produced approximately 2 times higher levels of ACOD1 than ipAM-Ls (Figure 6, A and B). To confirm that itaconate production is dependent on *ACOD1* in human iPSC-derived macrophages, we generated an *ACOD1-*deficient iPSC line using CRISPR gene editing. Two different disruptions of exon 3 were confirmed by Sanger sequencing (Supplemental Figure 5). ipMDM-L and ipAM-L cells were generated from *ACOD1*^–/–^ iPSCs, indicating that itaconate is not required for macrophage development in vitro. Immunoblotting for ACOD1 in LPS-stimulated *ACOD1*-KO macrophages of both types confirmed the absence of ACOD1 protein after LPS stimulation (Figure 6C).

To determine whether the difference in ACOD1 protein found in LPS-stimulated ipMDM-L and ipAM-L cells was reflected in itaconate production, and to confirm that *ACOD1*^–/–^ macrophages do not produce itaconate, we measured itaconate levels in macrophages by gas chromatography–mass spectrometry (GC-MS). ipMDM-L cells produced higher levels of itaconate compared with ipAM-L cells by a factor of 3- to 4-fold (Figure 6D). Itaconate levels in LPS-stimulated ACOD1*-*deficient macrophages were similar to unstimulated cells, as assessed by GC-MS (Figure 6D), and other Krebs cycle intermediates were not substantially affected by ACOD1 deletion (Supplemental Figure 7). To better understand the differences in itaconate production between human and murine macrophages, we stimulated murine BMDMs with LPS and observed 20-fold more itaconate than activated ipMDM-Ls and 50-fold more itaconate than activated ipAM-Ls (Figure 6D). These data demonstrate that human macrophages, despite strong induction of ACOD1 by LPS, produce substantially less itaconate than murine macrophages. iPSC-derived ipMDM-L cells produce more itaconate than alveolar-like macrophages, and CRISPR modification of iPSCs can be utilized to produce *ACOD1-*deficient macrophages.

### Attenuated itaconate production in Mtb-infected human macrophages.

LPS is a strong inducer of ACOD1 expression in both mouse (41, 51) and human macrophages (Figure 6A), but whether Mtb infection is an equivalently robust inducer of the enzyme is less clear. Prior data in murine BMDMs indicated that Mtb infection did induce mRNA encoding ACOD1, albeit to a lower level than LPS (52). We first assessed whether *ACOD1* transcript was induced in Mtb-infected ipMDM-L and ipAM-L cells by quantitating *ACOD1* mRNA in the RNA-Seq datasets presented above and found detectable *ACOD1* RNA by 4 hours of infection, which was maintained for at least 24 hours (Figure 6E). Infected ipMDM-L cells had a substantially higher induction of *ACOD1* mRNA at both time points compared with ipAM-L cells, which weakly induced the transcript (Figure 6E). However, although immunoblotting with anti ACOD1 antibodies again detected expression with LPS stimulation, Mtb infection at MOI of 3 did not induce detectable ACOD1 protein over either a 2-day or an extended 5-day infection (Figure 6F). Even at the nonphysiological MOI of 10, we did not observe ACOD1 protein, although LPS was still able to induce ACOD1 in Mtb-infected macrophages, indicating that Mtb was not actively inhibiting expression (Figure 6G). Measurement of itaconate in Mtb-infected macrophages revealed that itaconate levels did not rise above the level in uninfected cells during the first 4 hours of Mtb infection, but LPS stimulation still resulted in quantifiable levels (Figure 6H), thus paralleling the protein expression data. These data indicate that Mtb infection of human macrophages weakly induces the transcript encoding the ACOD1 protein and does not result in detectable itaconate production under the conditions tested.

### Itaconate deficiency impairs bacterial control by ipAM-Ls but not ipMDM-Ls.

**Figure 7:**
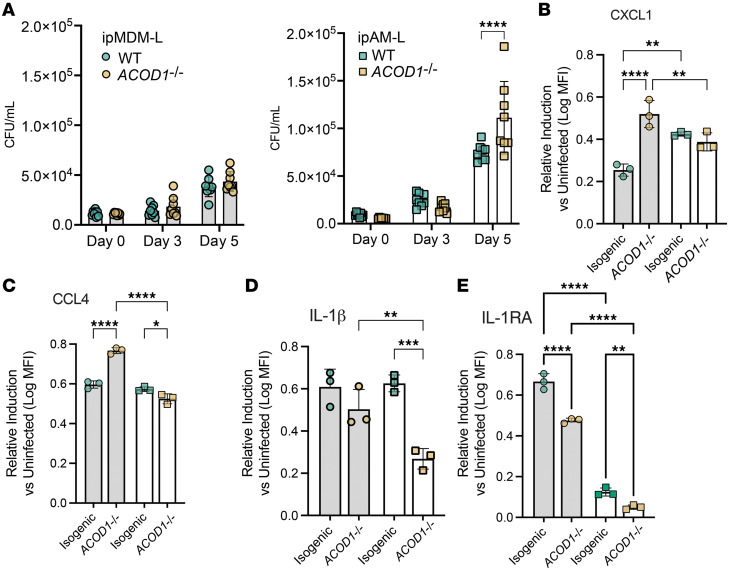
Macrophage type–specific roles of itaconate on Mtb control and inflammatory responses. (**A**) Mtb CFUs quantified from WT (green symbols) and *ACOD1^–/–^* (gold symbols) ipMDM-Ls (shaded bars) or ipAM-Ls (clear bars), infected at an MOI of 3, by day of infection (**B** and **C**). Relative concentration of CXCL1 (**B**) or CCL4 (**C**) produced by WT or *ACOD1* *^–/–^* ipMDM-Ls (shaded bars) and ipAM-Ls (clear bars) on day 3 of a Mtb infection. (**D** and **E**) Relative concentration of IL-1β (**D**) and IL-1RA (**E**) produced by WT and *ACOD1^–/–^* ipMDM-Ls and ipAM-Ls on day 3 of Mtb infection. ****P < 0.0001 by 2-way ANOVA with correction for multiple comparisons.

To assess the role of itaconate in macrophage control of Mtb, we assessed the capacity of *ACOD1-*deficient macrophages to control bacterial replication and produce cytokines and chemokines. *ACOD1* deficiency had no effect on bacterial control in ipMDM-L cells (Figure 7A). However, ipAM-Ls were more permissive of bacterial growth than the ipMDM-Ls on day 5, and *ACOD1* deficiency exacerbated this effect, indicating that this effect of itaconate on Mtb growth is specific to alveolar-like macrophages (Figure 7A). This result is surprising insofar as itaconate is postulated to exert a direct antimicrobial effect on intracellular bacteria, yet ipAM-L cells produce less ACOD1 protein and itaconate than ipMDM-L cells.

To verify the concentrations of itaconate required to inhibit Mtb growth in vitro, Mtb was grown in oleic acid-albumin-dextrose-catalase (7H9-OADC) or media with propionate as a sole carbon source (albumin-NaCl and 10 mM sodium propionate [7H9-ANP]) supplemented with itaconate in a range of concentrations from 5 μM to 15 mM. We found 3 mM itaconate in 7H9-OADC slowed Mtb growth, and 15 mM itaconate completely inhibited growth (Supplemental Figure 6A). Itaconate was more potent in ANP media, inhibiting growth at 625 μM (Supplemental Figure 6A). To exclude media acidification as a cause of growth inhibition by itaconate, which has been noted previously (53), we buffered itaconate-supplemented media with 100 mM 3-(N-morpholino)propanesulfonic acid (MOPS) and observed that Mtb was capable of replication in 7H9-OADC even with 15 mM itaconate (Supplemental Figure 6B). In buffered 7H9-ANP, Mtb growth was completely inhibited by 3 mM itaconate or greater and partially inhibited at 125 μM, indicating that the potency of itaconate for Mtb growth inhibition is modulated by both pH and carbon source (Supplemental Figure 6B).

### Macrophage subtype–specific effects of itaconate on inflammatory responses to Mtb.

Since itaconate has complex effects on mouse macrophage inflammatory pathways, we determined the effect of itaconate deficiency on Mtb-driven macrophage inflammatory responses by measuring chemokines and cytokines in Mtb-infected macrophage supernatants. Although *ACOD1-*deficient macrophages produced cytokines and chemokines in response to Mtb, CXCL1 and CCL4 levels were higher in *ACOD1-*deficient ipMDM-L cells than isogenic control cells, with no effect in ipAM-L cells (Figure 7, B and C). In contrast, IL-1β production was lower in *ACOD1-*deficient ipAM-L cells compared with isogenic controls, with no effect in ipMDM-L cells (Figure 7D). IL-1RA required ACOD1 for full expression in both macrophage types, although ipMDM-L cells more strongly induced this protein with infection (Figure 7E).

**Figure 8:**
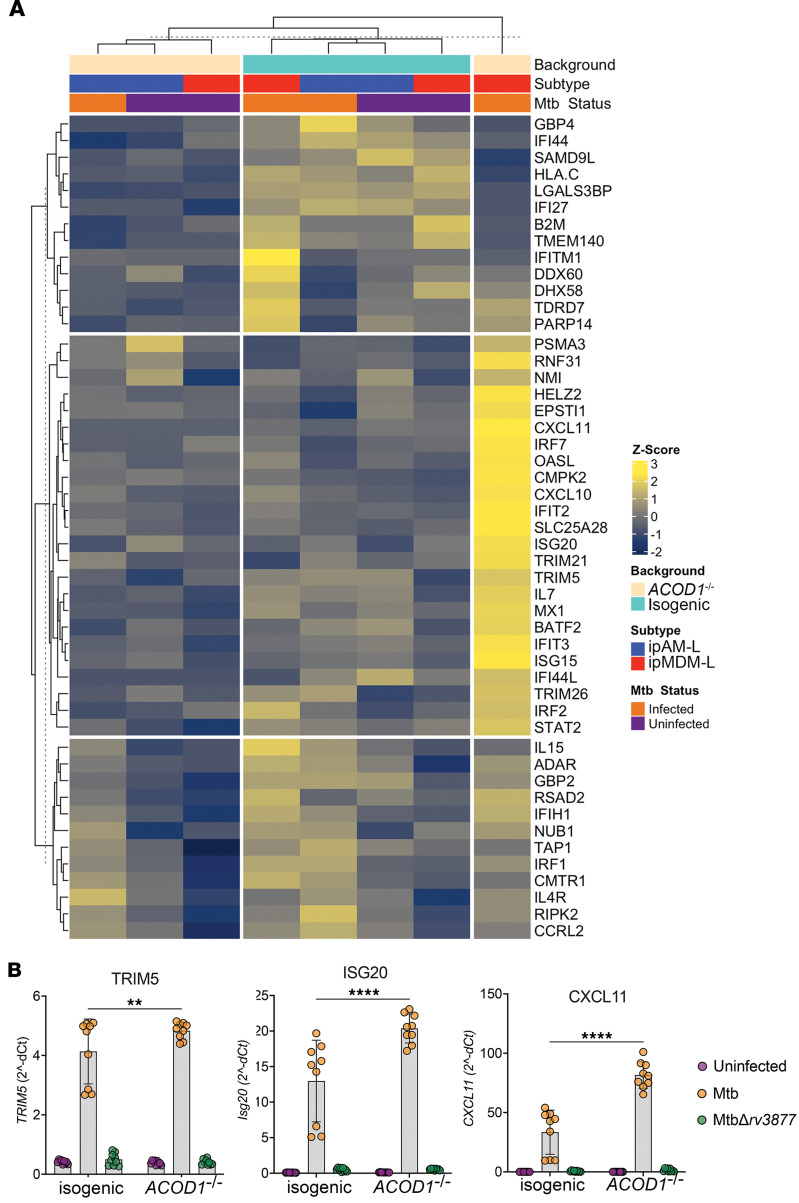
Itaconate negatively regulates the type I IFN response to Mtb in ipMDM-L but not ipAM-L cells. (**A**) Heatmap depicting *z* score of hallmark type I IFN gene expression in ipMDM-L and ipAM-L cells in the presence or absence of Mtb. Partitioning is based on k-means clustering. (**B**) Hyper-induction of the type I IFN pathway in itaconate-deficient macrophages is due to phagosomal permeabilization by Mtb *esx-1*. RT-qPCR quantifying *Trim5*, *Isg20*, and *Cxcl11* transcripts, normalized to GAPDH, in ipMDM-Ls in response to infection by WT Mtb or MtbΔ*rv3877* (lacking the *esx-1* secretion system) for 4 hours. **P < 0.01, ****P < 0.0001 by 2-way ANOVA with correction for multiple comparisons. Data are shown as mean ± SD.

To better understand how *ACOD1* deficiency affects the transcriptional response of human iPSC-derived ipMDM-L and ipAM-L cells, we performed RNA-Seq after 4 hours of Mtb infection and compared these data to prior experiments infecting murine *ACOD1-*deficient BMDMs (39). We focused on the TNF and type I IFN pathways based on the centrality of these macrophage responses to Mtb immunity (54–56) and prior reports that itaconate suppresses the TNF pathway in murine BMDMs (39). Mtb infection of WT ipMDM-L cells induced type I IFN response genes more vigorously than ipAM-L cells (Figure 8A). Similarly, infection of ipMDM-L cells broadly induced genes in the hallmark TNF/NF-κb pathway to a greater degree than in ipAM-L cells, whereas ipAM-L cells were relatively blunted in this response (Supplemental Figure 8A). In *ACOD1*-KO ipMDM-L cells, transcripts in both the TNF and type I IFN pathways were strongly overexpressed with tuberculosis infection compared with isogenic controls (Figures 8A and Supplemental Figure 8A). However, *ACOD1* deficiency had little effect on either the TNF or type I IFN pathways in ipAM-L cells (Figure 8A and Supplemental Figure 8A). These results demonstrate that *ACOD1* negatively regulates expression of both the TNF and type I IFN responses with Mtb infection, but that this function is limited to MDM-L cells and is not operative in AM-L cells. Prior data from *ACOD1-*deficient mouse BMDMs revealed similar hyper-induction of the TNF pathway, suggesting that murine BMDMs resemble ipMDM-L cells in response to Mtb. We reanalyzed this murine BMDM dataset, focusing specifically on type I IFN pathway genes, and found similar overexpression of this pathway (Supplemental Figure 8B), further buttressing the similarly between ipMDM-L cells and murine BMDMs with respect to the function of itaconate in suppressing macrophage responses to Mtb infection.

To confirm these results and determine the mechanism of type I IFN pathway hyper-induction in *ACOD1-*deficient cells, we quantitated the transcripts of the IFN-stimulated genes (ISGs) TRIM5, ISG20, and CXCL11 by RT-qPCR. *ACOD1*^–/–^ ipMDM-L cells accumulated higher levels of TRIM5, ISG20, and CXCL11 transcripts upon Mtb infection compared with isogenic ipMDM-L cells, confirming the pattern observed by RNA-Seq (Figure 8B). We postulated that itaconate may be modulating the induction of the ISG response that is derived through the previously established pathways of phagosomal permeabilization. To assess this, we infected ipMDM-L cells with MtbΔ*rv3877,* an Mtb strain with a deletion of *eccD1*, a core component of the Esx1 secretion system. Mtb lacking the Esx-1 secretion system cannot permeabilize the phagosome and does not induce the type I IFN pathway in macrophages (57–60). Infection with the MtbΔ*rv3877* strain completely abolished the hyperexpression of these IFN target genes in both WT and *ACOD1-*deficient ipMDM-L cells (Figure 8B). Taken together, these data indicate that the effects of itaconate on the macrophage ISG response proceeds through previously established pathways of phagosomal permeabilization.

## Discussion

Although Mtb is an obligate human pathogen that infects via droplet deposition in the terminal alveolus of the lung, studying the initial host cell, the AM, is difficult due to the challenge of obtaining sufficient cells from humans via bronchoalveolar lavage. Even when obtained via this method, these cells are short-lived and cannot be genetically manipulated. Although murine macrophage models have been widely used, less is known about how human macrophages interact with Mtb, and many of these studies focus on blood-derived MDMs. Given the distinct ontogeny of tissue-resident and blood-derived macrophages, the antimicrobial effector mechanisms these cells apply to Mtb may be distinct. To address some of these limitations, we have developed a genetically tractable human iPSC-derived macrophage model for alveolar and blood-derived macrophages and used this model to examine Mtb-macrophage interactions.

We found that iPSC-derived macrophages differentiated with GM-CSF resemble tissue-resident AMs based on expression of the hallmark AM transcription factors PPARG, KLF4, and CEBPB. Furthermore, we observed that iPSC-derived ipAM-L cells infected with Mtb respond similarly to primary human AMs and a newly published model of human alveolar-like macrophages derived from PBMCs (27, 42, 50). Both iPSC-derived ipAM-L and ipMDM-L cells produced chemokines and cytokines typical of Mtb-infected macrophages and induced genes related to type I IFN signaling, a hallmark of Mtb macrophage infection (55, 57). We assessed inter-donor variability and generally observed conserved responses to Mtb infection across donors. These data demonstrate that iPSC-derived macrophages are a useful model system for the interaction of Mtb with distinct human macrophage populations. Given the ability to derive these cells from iPSCs generated from peripheral blood, this system could also be used to interrogate clinically defined interindividual differences in tuberculosis control, especially those due to genetic etiologies manifested in macrophages (42). Another noteworthy feature of iPSC-derived macrophages is genetic tractability, which enables assessment of the contribution of specific genes to macrophage host defense, a task that is technically impractical when using primary human macrophages. The iPSC-derived macrophage system reported here provides an additional model for human AMs, in addition to the blood-derived AM system reported recently by Pahari et al. (50). Our data indicate that AM-like cells from these 2 protocols behave similarly with Mtb infection (Figure 3 and Supplemental Figure 4), with upregulation of inflammatory pathways that mimic those in human AMs collected from bronchoalveolar lavage. These 2 AM-like cell types offer complementary strengths and weaknesses depending on experimental purpose. The iPSC macrophage system offers genetic tractability, a feature that could be extended to a variety of genetic manipulations of the iPSC, including reporter genes and potentially genetic screening libraries. In addition, because the iPSC can be cryopreserved, this system is well-suited for repeated studies of a given donor or donor genotype because macrophages can be generated repeatedly from the same iPSC line without the repeated blood draws that would be required for the blood system. In contrast, the blood-derived AM-L system reported by Pahari et al. (50) offers much more rapid generation of AM-L cells from a simple blood draw, providing a less laborious method for generation of AM-L cells than the iPSC system. The blAM-L cell also can be directly compared with traditional MDMs prepared from the same donor. The iPSC system requires a more specialized and laborious experimental workflow (Supplemental Figure 1) than blood-derived AM-Ls. Blood-derived AM-L cells are therefore better suited to projects that compare large numbers of donors, which would be very onerous with the iPSC system due to its labor intensity and long generation time.

We leveraged the genetic tractability of iPSC-derived macrophages to interrogate the function of itaconate in human macrophages by disrupting *ACOD1*. Itaconate is an antimicrobial and immunomodulatory metabolite produced from the Krebs cycle intermediate aconitate through the inflammation-induced expression of *ACOD1*. Mouse macrophages produce millimolar (mM) quantities of itaconate (41), but the role of this metabolite in human macrophages is less clear. *ACOD1-*deficient mice, which cannot produce itaconate, are hypersusceptible to Mtb infection, but this susceptibility is due to pulmonary hyperinflammation rather than a macrophage cell–intrinsic antimicrobial defect (39).

Although we found that ipMDM-L cells produce more itaconate than ipAM-L cells, both macrophage types produced much less itaconate than murine BMDMs, despite robust induction of the ACOD1 enzyme. Itaconate deficiency also had distinct effects on Mtb control in different macrophage types. Lack of itaconate impaired bacterial control in alveolar-like macrophages but not ipMDM-L cells. In contrast, itaconate-deficient ipMDM-L cells controlled Mtb like WT cells, but hyper-induced the type I IFN and TNF pathways, an inflammatory response that was not observed in itaconate-deficient ipAM-L cells. The hyperinflammatory response in ipMDM-L cells, coupled with the lack of an effect on bacterial control, closely resembles observations in murine *ACOD1-*deficient BMDMs infected with Mtb (39) but differs from the function of itaconate in supporting type I IFN pathway induction in mouse BMDMs stimulated with LPS (61). A recent report examining the role of mouse BMDMs differentiated with either M-CSF or GM-CSF in *S*. *aureus* skin infection found opposing roles for the metabolite in suppressing or supporting TNF production (62), paralleling our findings in iPSC-derived human macrophage subtypes.

Our observed differences between itaconate-deficient ipAM-Ls and ipMDM-Ls are surprising because ipMDM-Ls produce more itaconate than ipAM-Ls, yet the latter have a more dramatic bacterial control defect, whereas the higher producers (ipMDM-Ls) have a purely inflammatory phenotype with itaconate deficiency. In both macrophage types, Mtb infection does not induce detectable ACOD1 protein, despite the robust induction of the protein by LPS, yet macrophages deficient in itaconate production still manifest Mtb control and inflammatory phenotypes. Prior data from *Salmonella* infection of macrophages indicates that the absolute quantity of itaconate produced is a poor correlate of macrophage-intrinsic microbial control due to subcellular compartmentalization of the metabolite. Macrophages that cannot deliver itaconate to the *Salmonella*-containing phagosomal compartment are defective for bacterial control, yet this defect does not affect cytosolic bacteria, and the absolute quantity of itaconate produced is not reduced, strongly indicating that cellular trafficking of itaconate is a major determinant of its direct antimicrobial effects in macrophages (63). Although we did not probe the underlying mechanisms of our observed cell type–specific effects of itaconate, the data may suggest that AMs channel itaconate efficiently to the phagosome to limit bacterial growth, and thereby potentially limit the inflammatory effects of itaconate, which are likely mediated by interaction with cytosolic receptors (64).

## Methods

### Sex as a biological variable.

The donor PBMCs from which iPSCs and macrophages were derived were from both male and female donors (*n* = 1 male, *n* = 2 female).

### Antibodies and cytokines.

The following antibodies were used: Alexa Fluor 488 anti-human CD206 (MRC1) Ab (Thermo Fisher Scientific, 564855), BV650 anti-human CD14 (BioLegend, 301836), APC/Cy7 anti-human CD45 (BioLegend, 304014), PE-Cy5 anti-human CD11c (BD Biosciences, 561692), PE/Cy7 anti-human CD11b (BioLegend, 301321), anti-ACOD1 antibody (Cell Signaling Technology, 77510S), HRP-conjugated human anti-vinculin (Cell Signaling Technology, 18799S), and IgG (H+L) goat anti-rabbit HRP (Invitrogen-Fisher, 656120). The following cytokines were used: recombinant human FGF-basic (154 aa) (Peprotech, 100-18B), recombinant human IL3 (Peprotech, 200-03-100 μg), recombinant human M-CSF (Peprotech, 300-25), and recombinant human GM-CSF (Peprotech, 300-03).

### Hematopoietic differentiation of human iPSCs into macrophages.

Generation of ipMDM-L and ipAM-L cells was modified from the methods described in Lachmann et al. (46). In brief, human iPSCs that were generated by reprogrammed PBMCs with Yamanaka factors were maintained and expanded on CF1-irradiated mouse feeder cells (Thermo Fisher Scientific, A34181) in ESC media (KO DMEM, Thermo Fisher Scientific,10829018) supplemented with 10% KO Serum Replacement (Thermo Fisher Scientific, 10828028), 1% penicillin-streptomycin (Thermo Fisher Scientific, 15140122), 100 μM β-mercaptoethanol (Thermo Fisher Scientific, 31350010), 1% nonessential amino acids (Thermo Fisher Scientific, 11140050), 2 mM L-glutamine (Thermo Fisher Scientific, 25-030-081) supplemented with 3–10 ng/mL basic fibroblast growth factor (bFGF). To induce embryoid body formation, iPSC colonies that were maintained in ESC without bFGF for 5 days were disrupted by treating with type IV collagenase (Thermo Fisher Scientific, 17104019, 250 U/mL), then cultured for 6 days in ESC supplemented with 10 μM rock inhibitor (Sigma-Aldrich, Y0503-5MG) while on an orbital shaker (100 rpm). To facilitate germ layer formation, aggregated iPSCs were manually disrupted after 6 hours of shaking, and Rock inhibitor was diluted 4-fold with fresh ESC after 3 days. Mature embryoid bodies were manually transferred into APEL II differentiation media (STEMCELL Technologies, 5270) supplemented with 1% penicillin-streptomycin, 5% protein-free hybridoma media (Thermo Fisher Scientific,12040077), 25 ng/mL human IL-3, and 50 ng/mL human M-CSF. After 2 weeks of maturation, suspension cells produced by the embryoid bodies were collected, and macrophage differentiation was induced by transferring these cells into macrophage differentiation media (RPMI 1640, Thermo Fisher Scientific, MT15040CV) supplemented with 10% heat-inactivated FBS and 2 mM L-glutamine) with either 100 ng/mL human M-CSF or 100 ng/mL human GM-CSF to produce ipMDM-Ls or ipAM-Ls, respectively. Macrophages were considered mature after being maintained in macrophage differentiation media for 5 days. Embryoid bodies were maintained in culture for up to 4 months once fully mature.

### Flow cytometry of macrophages.

iPSC-derived macrophages were detached by treatment with trypsin (TrypLE Express, Thermo Fisher Scientific, 12605-010) for 5 minutes before centrifugation at 400*g* for 5 minutes. The subsequent cellular pellet was resuspended in FACS buffer (PBS +0.5% BSA +1 mM EDTA). After blocking Fc receptors (Miltenyi Biotec,130-059-901) at 1:10 dilution for 10 minutes, the cells were washed with FACS buffer by centrifugation at 400*g* for 5 minutes, and immunostained in FACS buffer with the appropriate antibodies, diluted at a range of 1:50 to 1:200, and incubated for 30 minutes at 4°C. Data were acquired on either an ARIA III BD flow cytometer or a FACS Fortessa SORP instrument and analyzed with FlowJo. Dead cells and debris were excluded from the analysis using DAPI (1 μg/mL), side (SSC-A) and forward scatter (FSC-A) gating, and doublet exclusion using forward scatter width (FSC-W) against FSC-A. At least 5,000 cells were acquired for each condition.

### Cytology and imaging of iPSC-derived macrophages.

iPSC-derived macrophages were prepared for flow cytometry as described above. Between 1,000 and 5,000 cells were then sorted into 1.5 mL Eppendorf tubes, which had been precoated for 2 hours with PBS with 20% FBS (200 μL of FBS at room temperature). The sorted cells were loaded into Cytofunnels (Thermo Fisher Scientific, BMP-CYTO-DB25) attached to super-frost slides (Thermo Fisher Scientific, 12-550-15) before centrifugation with Cytospin 3 (Thermo Shandon) at 800 rpm for 10 minutes under medium acceleration. The slides were air-dried for at least 30 minutes and fixed for 10 minutes in 100% methanol (Thermo Fisher Scientific, A412SK-4). Methanol-fixed cells were stained in May-Grünwald solution (Sigma-Aldrich, MG500-500 mL) for 10 minutes and washed twice with UltraPure distilled water (Thermo Fisher Scientific, 10977-023). The cells were then stained for 10 minutes with 10% Giemsa (Sigma-Aldrich, 48900-500 mL-F) diluted in UltraPure distilled water. After this stain, cells were washed twice with UltraPure distilled water and left to air-dry overnight. The slides were mounted with Entellan New (MilliporeSigma, 1079610100) after air-drying, and representative pictures were taken using an Axio Lab.A1 microscope (Zeiss) under an N-Achroplan 100×/01.25 objective.

### Infection of macrophages.

Low-passage Mtb strain Erdman was used for infected ipMDM-L and ipAM-L cells. Mtb was grown to log phase in 7H9 Middlebrook medium supplemented with 0.5% glycerol, 0.05% Tween-80, and 10% OADC while maintained at 37°. To prepare a single-cell mycobacterial suspension, Mtb was washed by centrifugation twice with PBS supplemented with 0.05% Tween-80 (PBST80), followed by centrifugation at 200*g* for 10 minutes to remove aggregated bacteria. Based on the OD_600_ of the final supernatant, a conversion factor of OD_600_ of 1.0, equating to approximately 5 × 10^8^ CFU/mL, was used to prepare the inoculum. Macrophages were infected at a target MOI of 3, unless stated otherwise. After approximately 4 hours, extracellular Mtb was removed by washing once with PBS. Infected macrophages were maintained in macrophage differentiation media at 37°C and 5% CO_2_ for up to 5 days.

### Evaluation of Mtb-infected macrophages.

The supernatant of Mtb-infected cells was filtered twice through a 0.22 μm filter before storage at –80°C. Supernatants were then used to quantify cytokine and chemokine levels by Luminex, according to the manufacturer’s instructions. To assess bacterial replication control, infected macrophages were lysed by incubating with sterile water supplemented with 0.01% Triton X-100 at room temperature for 20–40 minutes before pipetting was used to mechanically lyse cells. Cell lysates were diluted with PBST_80_ and cultured on 7H10 Middlebrook agar supplemented with 0.5% glycerol and 10% OADC for 3 weeks at 37°C before quantification. RNA was isolated from macrophages by washing once with PBS followed by lysis and RNA preservation with TRIzol, according to the manufacturer’s instructions.

### RNA isolation and RNA-Seq.

Phase separation in cells lysed in 1 mL TRIzol reagent (Thermo Fisher Scientific, 15596018) was induced with 200 μL chloroform. RNA was extracted from 350 μL of the aqueous phase using the miRNeasy Micro kit (QIAGEN, 217084) on the QIAcube Connect (QIAGEN) according to the manufacturer’s protocol. Samples were eluted in 34 μL or 15 μL RNase-free water. After RiboGreen quantification and quality control with an Agilent BioAnalyzer, 29–100 ng of total RNA with RNA integrity number values of 6.5–9.8 underwent polyA selection and TruSeq library preparation according to instructions provided by Illumina (TruSeq Stranded mRNA LT kit, RS-122-2102), with 8 cycles of PCR. Samples were barcoded and run on a NovaSeq 6000 in a PE50 run, using the NovaSeq 6000 SP reagent kit (100 cycles) (Illumina). An average of 59 million paired reads was generated per sample. Ribosomal reads represented 1.1%–2% of the total reads generated, and the percentage of mRNA bases averaged 78%.

### RNA-Seq of ACOD1-deficient macrophages.

After RiboGreen quantification and quality control using the Agilent BioAnalyzer, 2 ng total RNA with RNA integrity numbers ranging from 4.1 to 10 underwent amplification using the SMART-Seq v4 Ultra Low Input RNA kit (Clontech, 63488), with 12 cycles of amplification. Subsequently, 7 ng of amplified cDNA was used to prepare libraries with the KAPA Hyper Prep kit (Kapa Biosystems, KK8504) using 8 cycles of PCR. Samples were barcoded and run on a NovaSeq 6000X in a PE100 run, using the NovaSeq 6000 S4 reagent kit (200 cycles) (Illumina). An average of 34 million paired reads were generated per sample. Ribosomal reads represented 0.04%–0.14% of the total reads generated, and the percentage of mRNA bases averaged 93%. RNA-Seq data have been deposited in NCBI’s Sequence Read Archive (SRA) under BioProject accession PRJNA1293194.

### RNA-Seq data analysis.

DEGs were determined as previously described by Campo et al (27). All 3 datasets were re-quality filtered, normalized, and modeled together. Briefly, sequences were quality trimmed and aligned to the human genome (GRCh38) using STAR. Alignments were quality filtered, and then reads were quantified using Rsubread. Counts were filtered to protein-coding genes with a minimum of 0.1 counts per million (CPM) in at least 3 samples and normalized via trimmed mean of means (TMM) and log_2_ CPM. DEGs were defined as FDR less than 0.05 for Mtb versus media in cells within a single mixed-effects model with interaction term blocked by donor, ~cell+Mtb+cell:Mtb+(1|donor), in kimma (65). For comparison of baseline gene expression of ipAM-L versus ipMDM-L cells and ex vivo AMs versus MDMs, normalization and differential gene expression were performed in DESeq2 (66). Gene set enrichment analysis (GSEA) was performed using gene-level estimates (log_2_ fold changes) of Mtb versus media within cell types against the Hallmark gene sets in Broad’s Molecular Signatures Database (MSigDB) (67) using SEARchways (68). Significant gene sets were defined at FDR less than 0.2.

### ACOD1 immunoblotting.

Mature iPSC-derived macrophages were treated with LPS (100 ng/mL) for either 4 or 6 hours, Mtb (MOI 10), or Mtb (MOI 10) and LPS (100 ng/mL) for 4 hours. Cells were washed once with PBS then treated with trypsin and incubated at 37°C for 5 minutes. After this incubation, trypsin was neutralized with 2× volume of cold macrophage differentiation media, and cells were detached by pipetting and scraping the bottom of the well. Cells were then centrifuged at 400*g* for 5 minutes before the cellular pellet was resuspended in cold lysis buffer (Tris-HCl pH 7.4, NaCl, EDTA, Triton X-100, Na_3_VO_4_, phosSTOP EASYPACK, Pefabloc, and EDTA-free complete protease inhibitor cocktail) and incubated for 20 minutes on ice before centrifuging at 16,000*g* for 10 minutes at 4°C. The resulting supernatant was then transferred to a new tube and diluted 1:1 with 2× loading dye (20% glycerol, 125 mM Tris HCl pH 6.8, 4% SDS, 0.2% bromophenol blue, 1× DTT) and incubated at 85°C for 1 hour. Protein was then separated on a 4%–12% NuPAGE bis-tris polyacrylamide gel. Equal loading was confirmed with commercially available HRP-conjugated human anti-vinculin antibody at a 1:1,000 dilution. ACOD1 was detected with human anti-ACOD1 antibody at a 1:1,000 dilution. Blots were blocked and probed in 5% Omniblok nonfat milk in 1× PBST_20_ (PBS + 0.01% Tween 20). Blots were imaged with iBright FL1000, and quantification of band intensity was conducted with Fiji.

### Generation and validation of ACOD1-KO iPSC line.

The CRISPR sgRNA target was designed using the web resource (https://www.benchling.com/crispr). The sgRNA target sequence ACAAAAGCAGCATATGTGGG was cloned into the pSpCas9(BB)-2A-Puro (PX459) V2.0 vector (Addgene plasmid 62988) to make the gene targeting construct. To knockout ACOD1, 170A iPSC lines were dissociated using Accutase (Innovative Cell Technologies) and electroporated using Amaxa 4D-Nucleofector (Lonza) following the manufacturer’s instructions. The cells were then seeded, and 4 days later, iPSCs were dissociated into single cells with Accutase and subcloned. Around 10 days later, individual colonies derived from single cells were picked, mechanically disaggregated, and replated into 2 individual wells of 96-well plates. A portion of the cells was analyzed by PCR and Sanger sequencing using amplification primers (forward-AGGGCTTCTATCTGTGGCAA, reverse-ACTTGAGAGAAACTGGCCCC) spanning to the CRISPR editing site and sequenced by Sanger sequencing using primer CCTTCCATCTTCTTTCCTCTGC. Biallelic frameshift mutants were chosen as KO clones. WT clonal lines from the same targeting experiments were included as controls.

### Detection of itaconate by GC-MS.

Mature iPSC-derived macrophages were seeded in 6-well plates treated with tissue culture. These cells were then treated with either LPS (100 ng/mL), Mtb (MOI 10), or LPS (100 ng/mL) and Mtb (MOI 10) together for 4 hours. At collection, metabolites were extracted with 450 μL ice-cold 80% methanol containing 2 μM deuterated 2-hydroxyglutarate (d-2-hydroxyglutaric-2,3,3,4,4-d5 acid [d5-2HG]), while maintained on dry ice. After at least an overnight incubation at –80°C, lysates were collected and centrifuged at 21,000*g* for 20 minutes at 4°C to remove protein. Samples that were treated with Mtb were then filtered twice through a 0.22 μm filter. All extracts were further processed using GC-MS as described below.

Metabolite extracts were dried in an evaporator (Genevac EZ-2 Elite) and resuspended by incubating with shaking at 30°C for 2 hours in 50 μL of 40 mg mL-1 methoxamine hydrochloride in pyridine. The metabolites were further derivatized by adding either 25 μL of N-methyl-N-(trimethylsilyl) trifluoroacetamide (Thermo Fisher Scientific) and 80 μL ethyl acetate (Sigma-Aldrich), or 80 μL MSTFA, and then incubated at 37°C for 30 minutes. The samples were analyzed using the Agilent 7890A gas chromatograph coupled to an Agilent 5975C mass selective detector. The gas chromatograph was operated in split-less injection mode with constant helium gas flow at 1 mL/min-1; 1 μL of derivatized metabolites was injected onto an HP-5ms column, and the gas chromatograph oven temperature increased from 60°C to 290°C over 25 minutes. Peaks representing compounds of interest were extracted and integrated using MassHunter v.B.08 (Agilent Technologies) and then normalized to both the internal standard (d5-2HG) peak area and the protein content of duplicate samples as determined using the BCA assay (Thermo Fisher Scientific). Steady-state metabolite pool levels were derived by quantifying the following ions: d5-2HG, 354 m/z; and itaconate, 259 m/z. Peaks were manually inspected and verified relative to known spectra for each metabolite.

### Determination of Mtb sensitivity to itaconate.

Mtb*-*Erdman was grown in liquid 7H9 media supplemented with 0.05% Tween_80_ and either 10% OADC or 10% ANP. Itaconic acid (Sigma-Aldrich, I29204) was then added to these liquid media at a range of 30 mM down to 2 μM. These media were also prepared with 100 mM MOPS supplementation to prevent a pH change due to the itaconate addition. Once Mtb*-*Erdman reached an OD_600_ of 0.4–0.7, the bacterial culture was then diluted to an OD_600_ of 0.06 and mixed at a 1:1 ratio with media containing itaconate. This resulted in a final OD_600_ of 0.03 and an itaconate range of 15 mM to 1 μM. The OD_600_ of the culture was then assessed every 2–3 days to quantify bacterial growth.

### RT-qPCR assessment of type I IFN genes.

ipAM-L and ipMDM-L cells derived from either WT or *ACOD1-*deficient iPSCs were infected with either Mtb-Erdman or MtbΔRv3877 at an MOI of 3 as stated previously. After a 4-hour incubation with Mtb, the media was removed, and the well was washed with PBS before being lysed with TRIzol. RNA was purified from the TRIzol-preserved samples with Direct-zol RNA MiniPrep (Thermo Fisher Scientific, 50-444-622), according to the manufacturer’s instructions. cDNA was generated from these samples with a Maxima First Strand cDNA Synthesis kit (Thermo Fisher Scientific, K1672). To quantify TRIM5, ISG20, and CXCL11 induction, a TaqMan fast advance master mix for qPCR was used (Thermo Fisher Scientific, 44-445-57). For this assay, we used predesigned primers and probes from Thermo Fisher Scientific. This assay used TRIM5 (Thermo Fisher Scientific, Hs01552558_m1) conjugated to 6-Carboxyfluorescein (FAM), ISG20 (Thermo Fisher Scientific, Hs00158122_m1) conjugated to FAM, or CXCL11 (Thermo Fisher, Scientific Hs00171138_m1) conjugated to FAM. As an internal reference, GAPDH (Thermo Fisher Scientific, Hs02786624_g1) conjugated to 2′-chloro-7′-phenyl-1,4-dichloro-6-carboxyfluorescein (VIC) was used. To assess for induction of these transcripts, ΔΔCT was normalized to GAPDH for each gene.

### Statistics.

The statistical tests used for all data are specified in the figure legends. Please see the RNA-Seq section for statistical tests used. In instances where multiple donors, multiple genetic backgrounds, or multiple time points were compared, a 2-way ANOVA with correction for multiple comparisons was utilized. When a single variable was being assessed, an unpaired, 2-tailed *t* test was utilized. *P* values of less than 0.05 were considered significant.

### Study approval.

Study participants gave written informed consent for blood collection at the GHESKIO Center Port Au Prince Haiti under IRB protocols approved by the GHESKIO and Weill Cornell Medicine IRBs (for donors 1 and 2, siblings) or by the Health Research Ethics Committee of Stellenbosch University (N16/03/033 and N16/03/033A, for donor 3).

### Data availability.

RNA-Seq data has been deposited in SRA under BioProject accession PRJNA1293194. Primary data for the figures are provided in the Supporting Data Values file in the supplemental materials.

## Author contributions

ASK, TL, AX, ALN, EEK, MM, TRH, LWSF, MAJJ, DWF, FG, and MSG designed the research studies. ASK, TL, ATR, AX, MD, OL, TZ, JAB, AZ, and ALN conducted experiments. ASK, TL, ATR, KADM, AX, JMB, ALN, TRH, TZ, LWSF, FG, and MSG analyzed the data. SBD and JLC provided reagents. ASK, TL, SBD, JLC, FG, and MSG wrote the manuscript.

## Funding support

This work is the result of NIH funding, in whole or in part, and is subject to the NIH Public Access Policy. Through acceptance of this federal funding, the NIH has been given a right to make the work publicly available in PubMed Central.

- NIH grants U19AI135990 (Human Pathogen Mapping Initiative), U19AI162568 (Tri-I TBRU), and R01AI124349.
- NIH/National Cancer Institute Cancer Center Support grant P30 CA008748.

## Supplementary Material

Supplemental data

Unedited blot and gel images

Supporting data values

## Figures and Tables

**Table 1:**
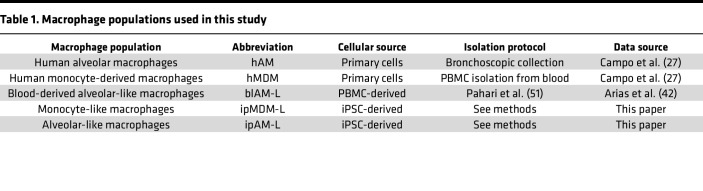
Macrophage populations used in this study

## Version 1 03/09/2026

Electronic publication
