# Supplementary figures and images for: Human iPSC-derived alveolar macrophages reveal macrophage subtype functions of itaconate in M. tuberculosis defense
Source: JCI Insight. 2026 Mar 9;11(5):e198342. doi: 10.1172/jci.insight.198342 (PMC13041677; doi:10.1172/jci.insight.198342)

A

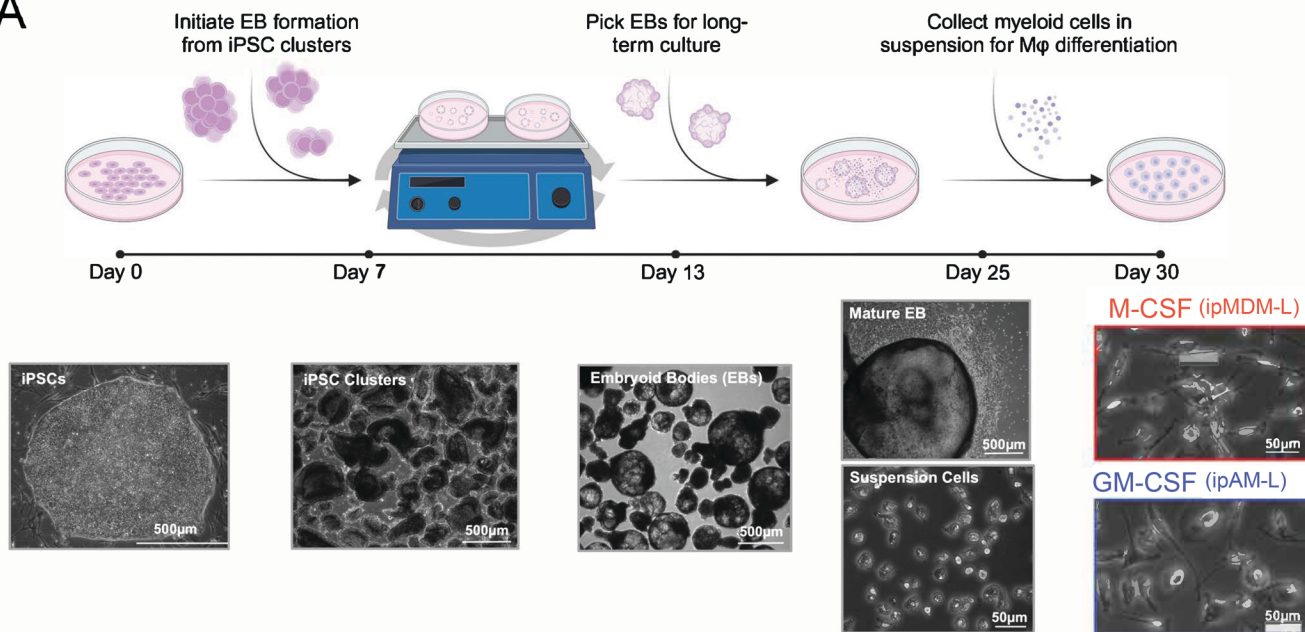

B

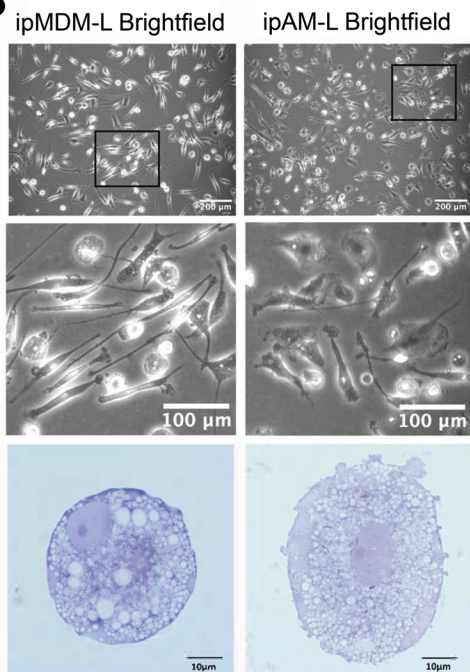

Figure S1

A

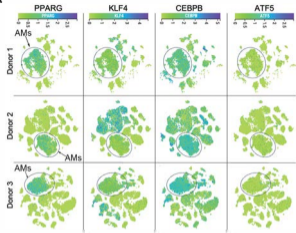

B

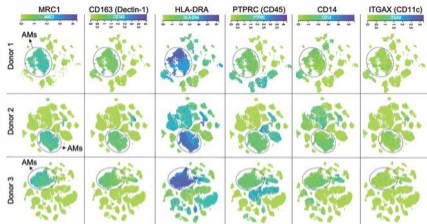

C

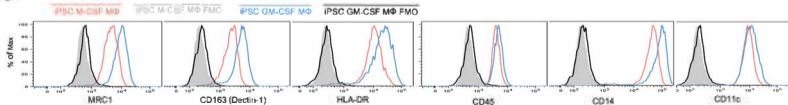

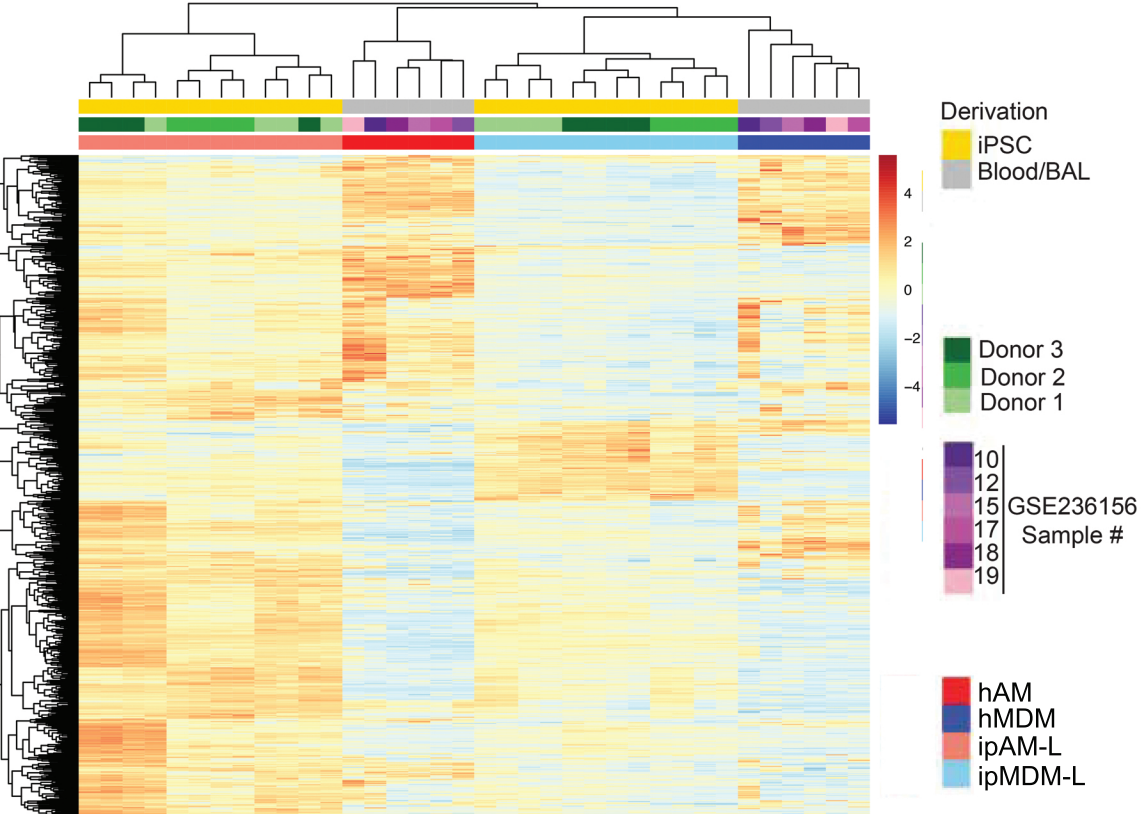

Figure S3

Figure S4

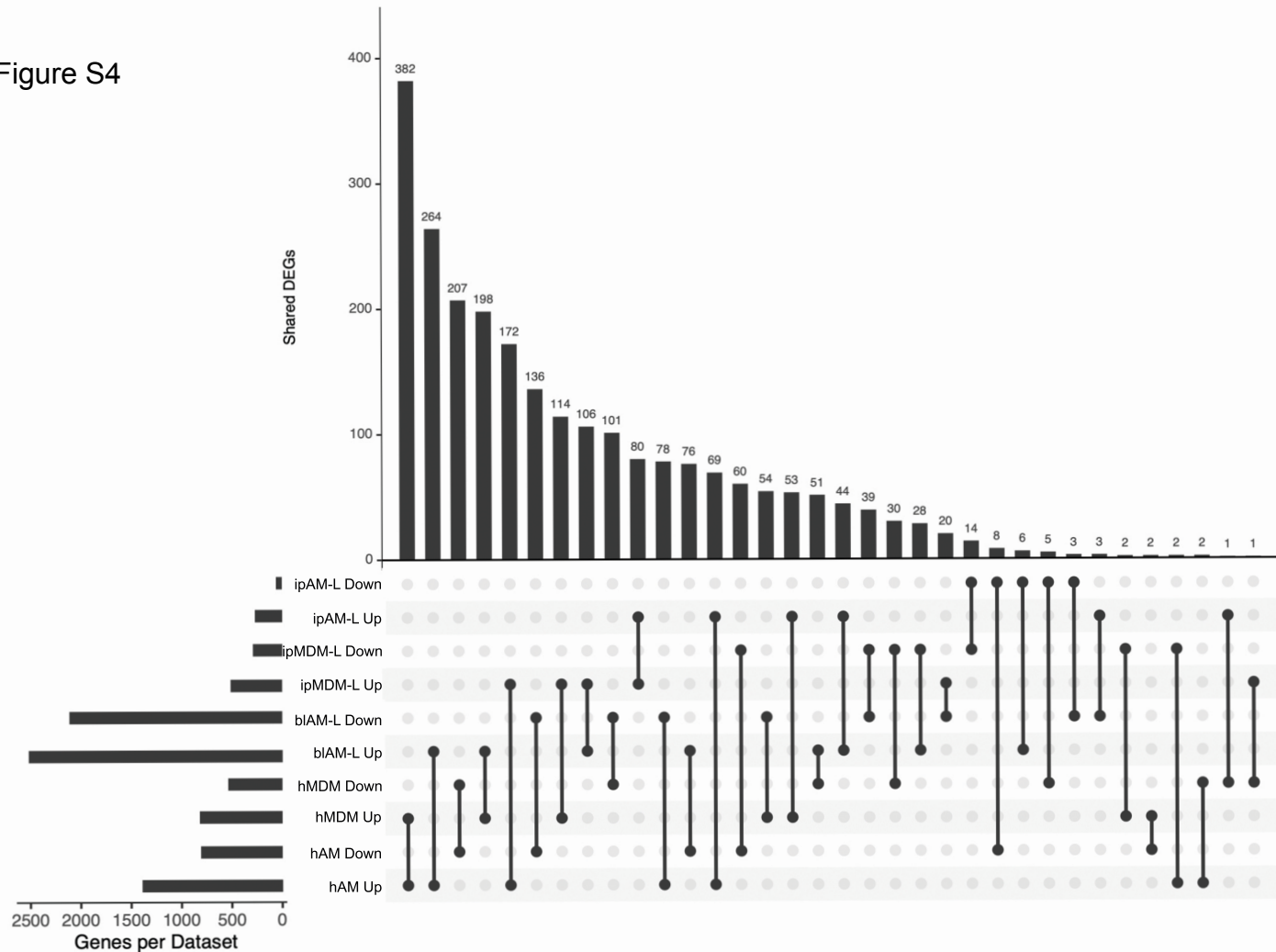

A

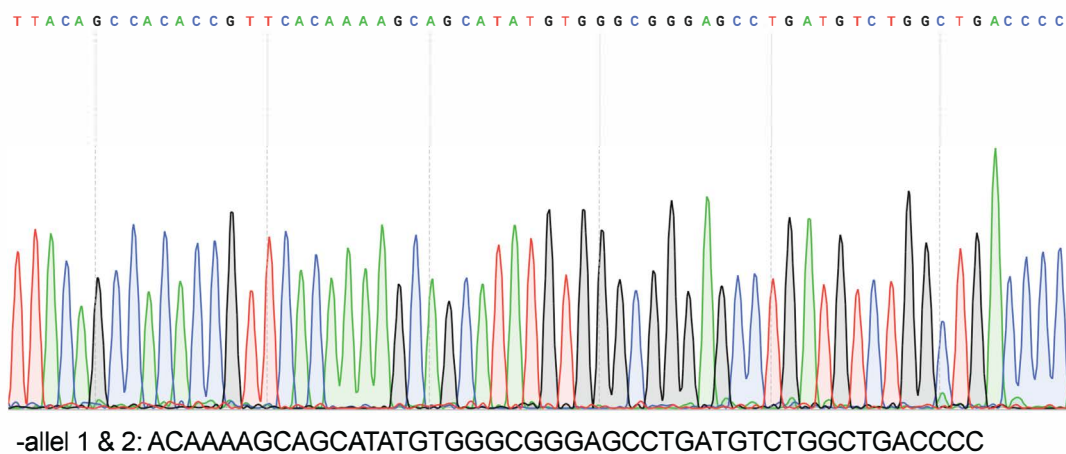

B

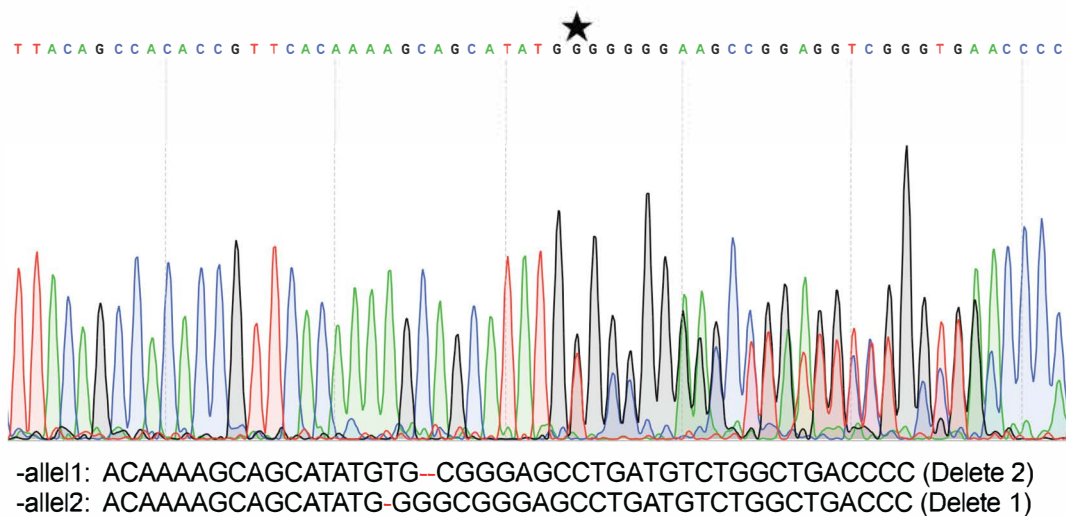

C

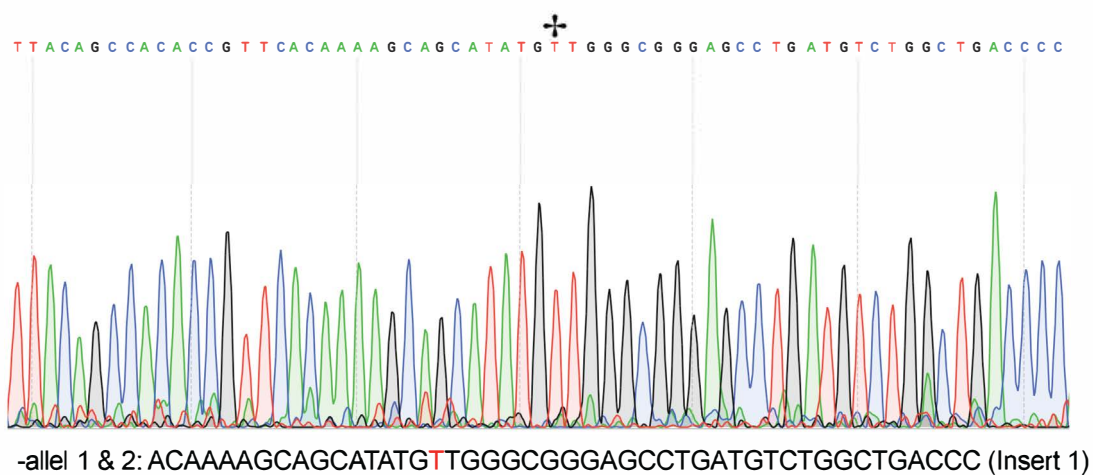

Figure S5

A

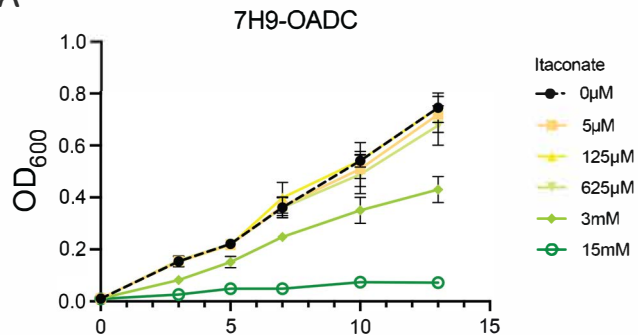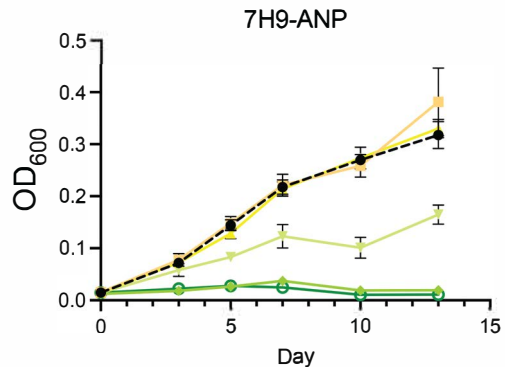

B

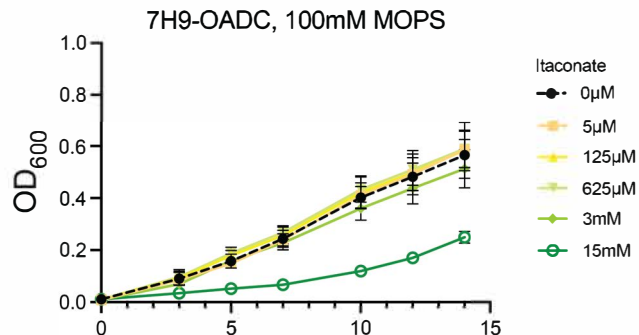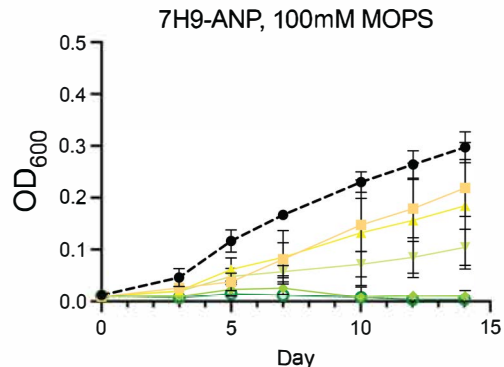

Figure S6

**A**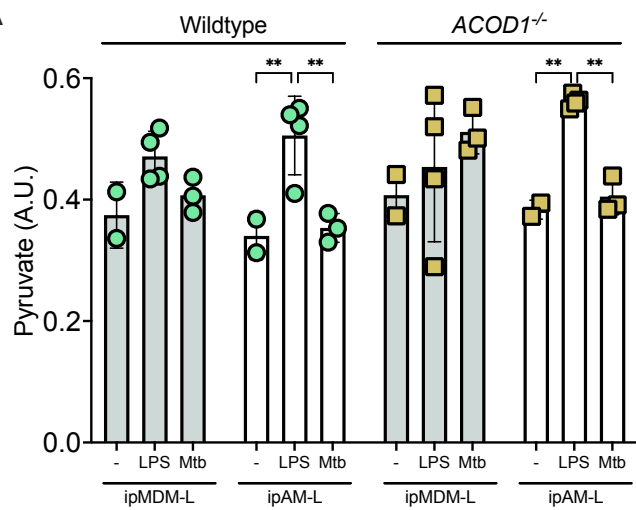**B**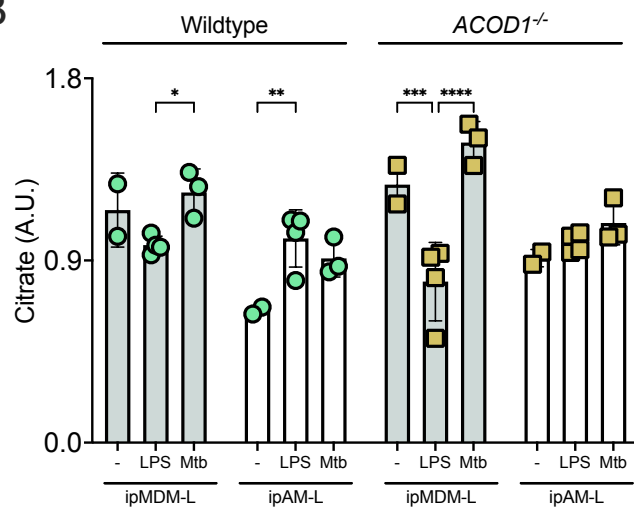**C**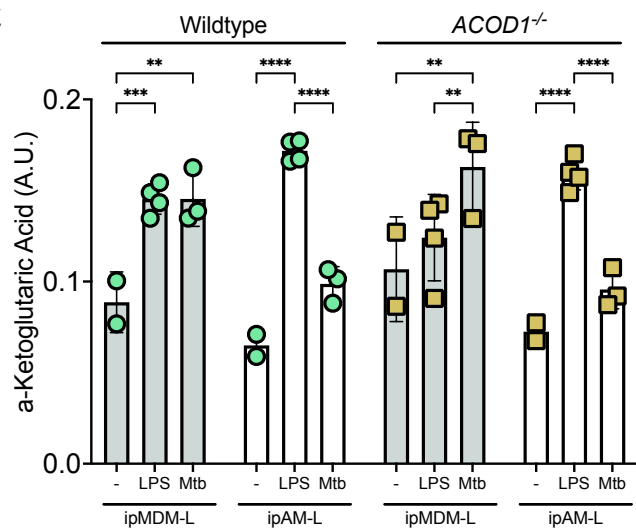**D**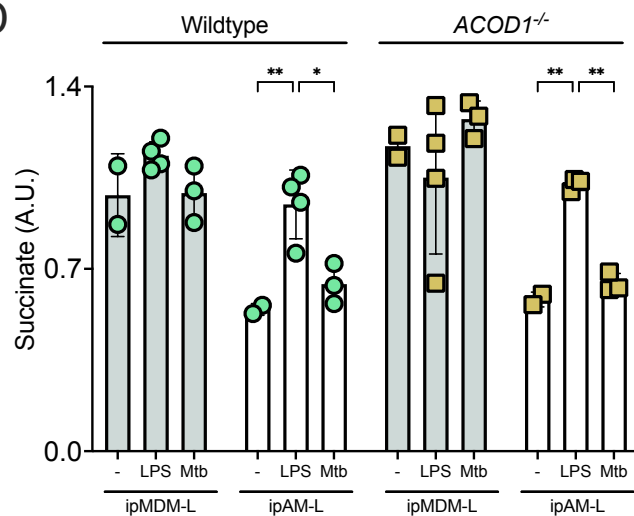**E**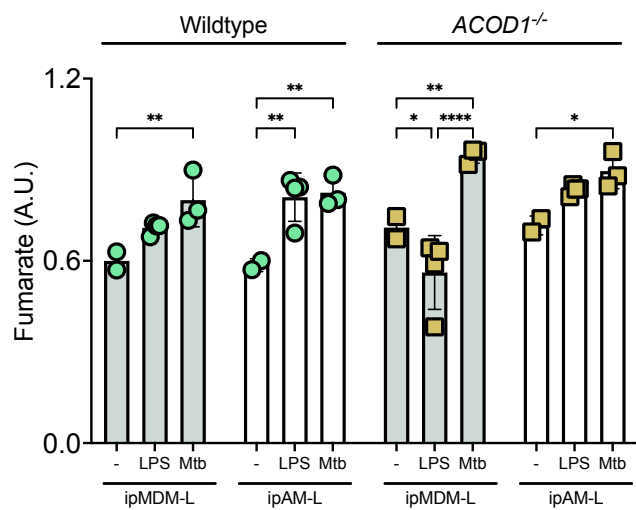**F**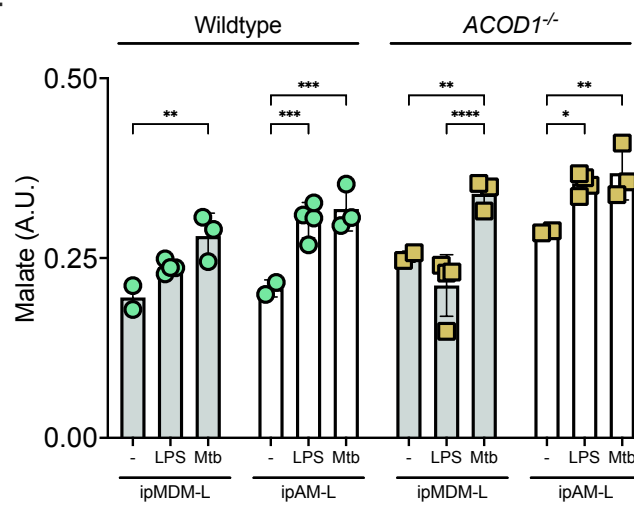

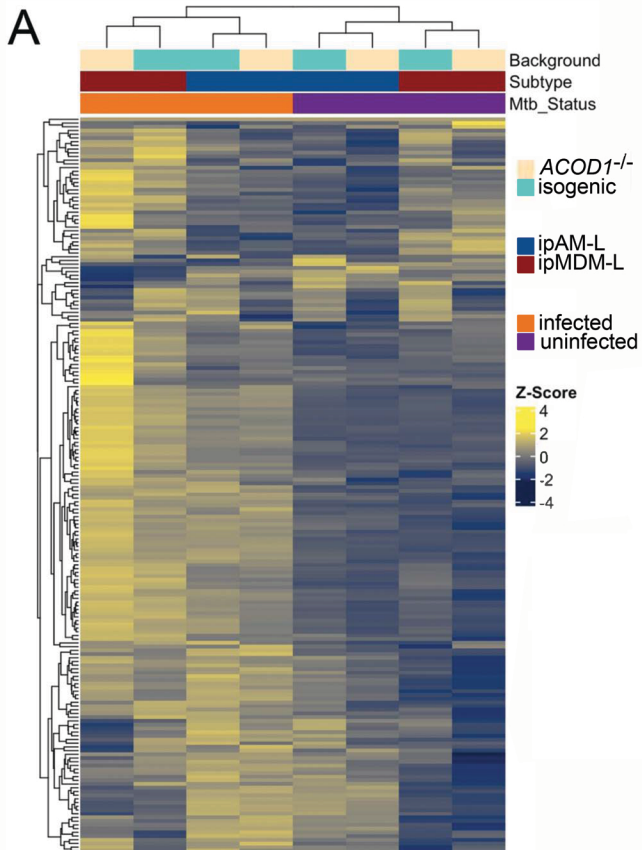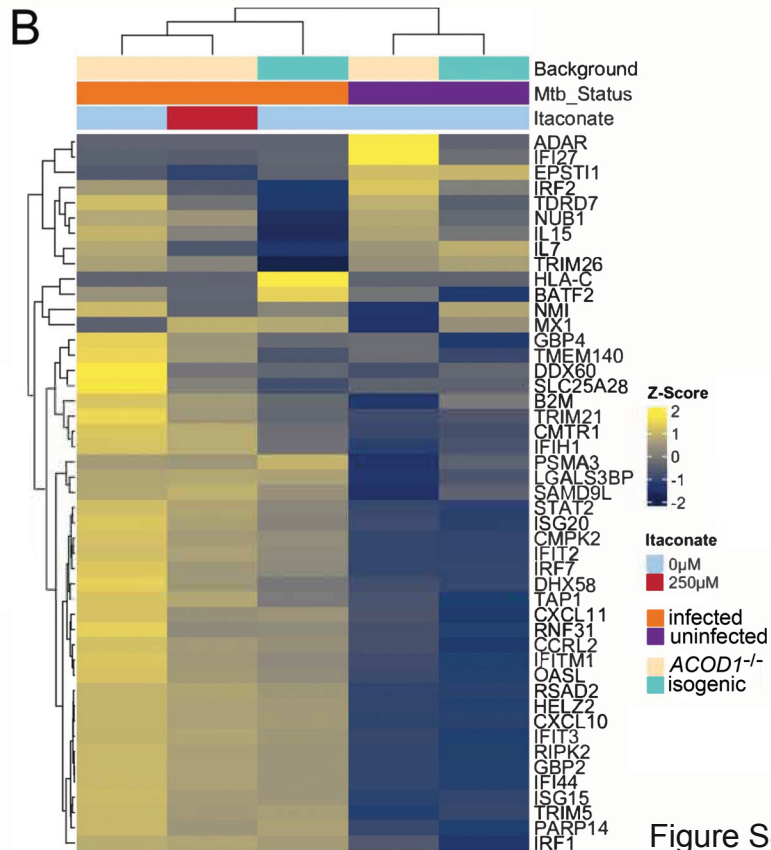

Figure S8

Supplement: Supplemental data [file jciinsight-11-198342-s157.pdf]

6A

Vinculin

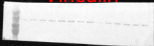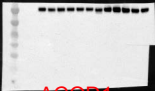

ACOD1

# Panel 6C

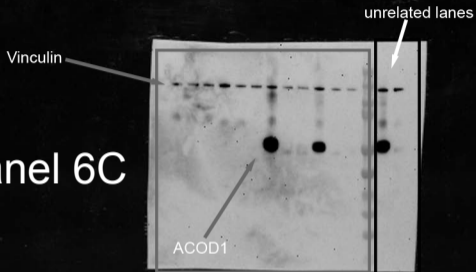

Vinculin

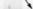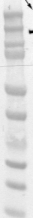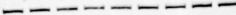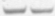

ACOD1

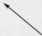

Fig 6F Top

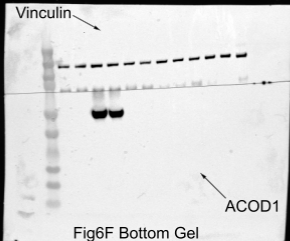

Fig 6G

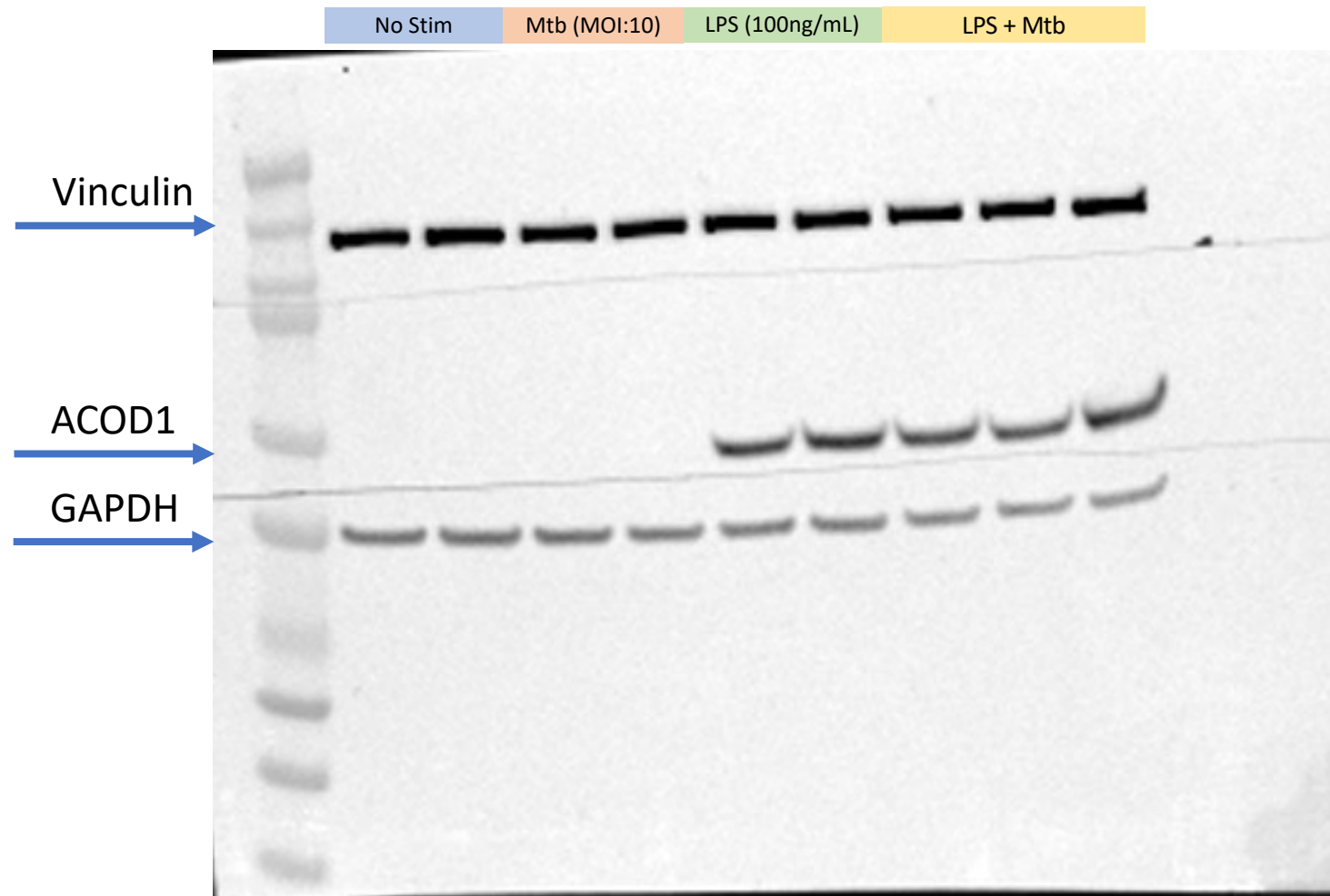

Supplement: Unedited blot and gel images [file jciinsight-11-198342-s158.pdf]
